# Supplementary material for: Industrial brewing yeast engineered for the production of primary flavor determinants in hopped beer
Source: Nat Commun. 2018 Mar 20;9:965. doi: 10.1038/s41467-018-03293-x (PMC5861129; doi:10.1038/s41467-018-03293-x)
Supplement: Supplementary file 1 — Supplementary Information [file 41467_2018_3293_MOESM1_ESM.docx]

Industrial brewing yeast engineered for the production of primary flavor determinants in hopped beer

Denby and Li *et al.*


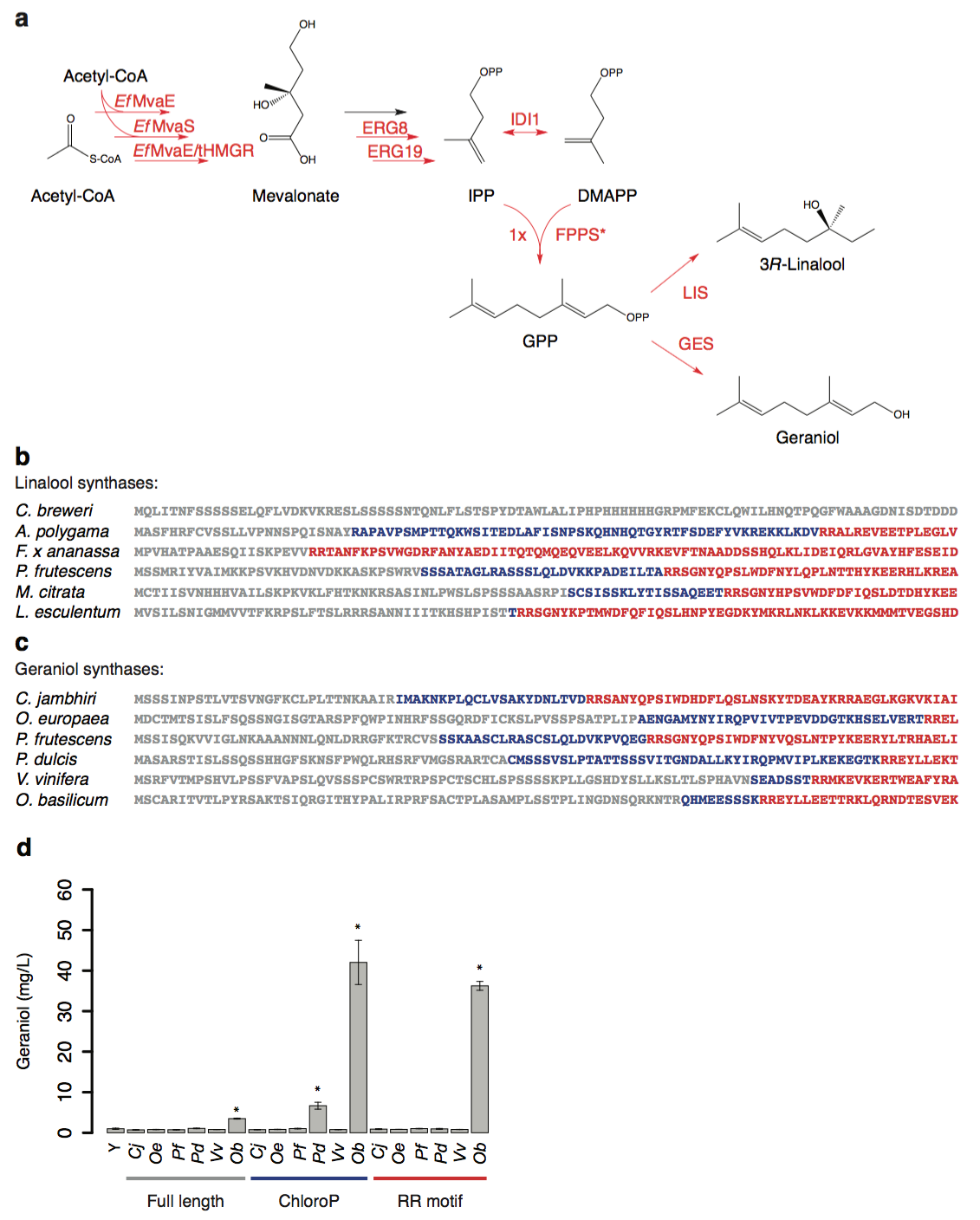


**Supplementary Figure 1** Screening activity of full-length and PTS-truncated linalool and geraniol synthases.

(**a**) Schematic of genetic modifications incorporated into GPP hyper-producing strain (derived from CEN.PK2-1D) used for screen. Red arrows correspond to genetic modifications aimed to increase flux towards linalool/geraniol. Details on genetic modifications, strain construction procedure, and constructs used to incorporate genetic modifications can be found in Supplementary Tables 7 and 8 and Online Methods. (**b,c**) Sequences of N-terminal amino acids for linalool and geraniol synthases, respectively. Red text indicates the PTS-truncated protein sequence as predicted by the RR-heuristic method, blue text indicates additional sequence predicted by ChloroP, and gray text indicates additional sequence corresponding to the full-length peptide. The linalool synthase from *C. breweri* does not contain a RR motif and is not predicted to contain a PTS by ChloroP. (**d**) Geraniol titers produced by cells overexpressing monoterpene synthase genes (expressed from high-copy plasmids) as measured by GC/MS. Plant species of origin are indicated by initials (*Citrus jambhiri*, *Cj*; *Olea europaea*, *Oe*; *Perilla frutescens*, *Pf*; *Phyla dulcis*, *Pd*; *Vitis vinifera*, *Vv*; *Ocimum basilicum*, *Ob*), Y indicates the control strain, full-length peptides and PTS-truncated peptides predicted by either ChloroP or the RR-heuristic method are indicated by colored bars underlining the plots: grey, blue and red, respectively. Error bars correspond to mean ± standard deviation of three biological replicates. Asterisks indicate statistically significant increases in monoterpene production compared with the control strain (Y) as determined by a t-test using p-value < 0.025. The full-length *O. basilicum* geraniol synthase (*Ob*) was identified as sufficiently active to allow for monoterpene production at levels characteristic of commercial beer, with the truncated peptide predicted by either method resulting in a 10-fold increase in production. The authors note that this geraniol synthase has been used in previous metabolic engineering efforts.


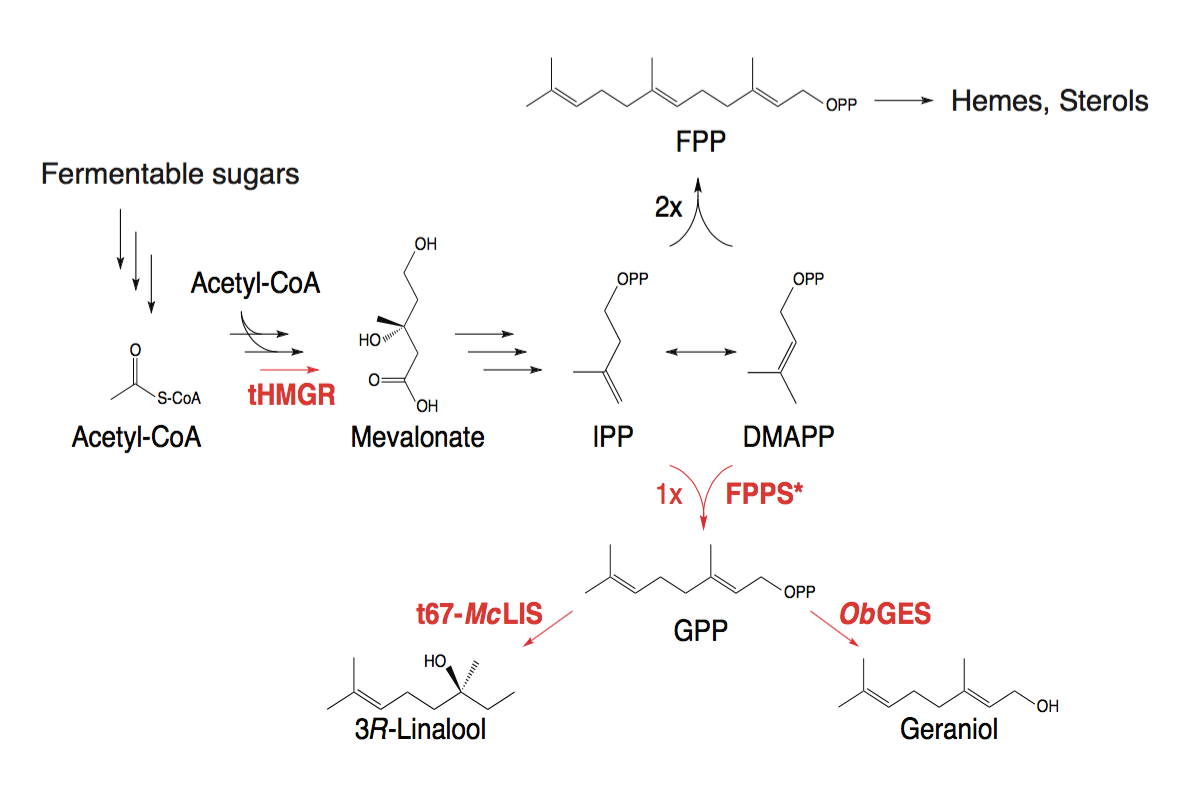


**Supplementary Figure 2** Schematic of genetic modifications incorporated into brewer’s yeast strains.

Red arrows correspond to genes that were modulated to tune linalool/geraniol production. Details on genetic modifications, strain construction procedure, and constructs used to incorporate genetic modifications can be found in Supplementary Tables 9-11 and Online Methods.


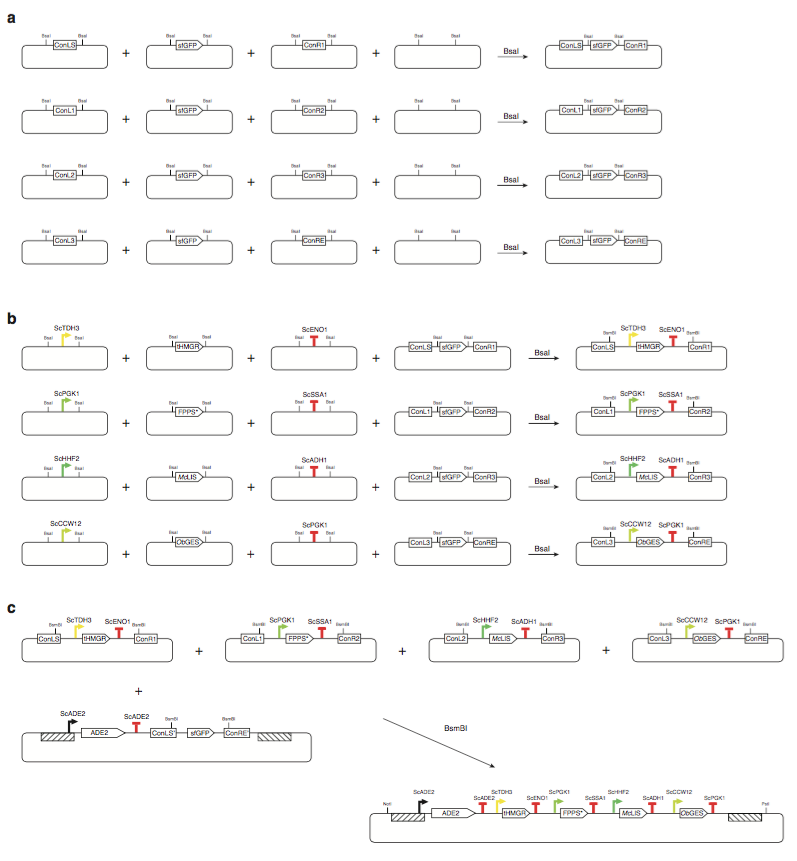
**Supplementary Figure 3** Schematic illustrating the construction of a repair template for Cas9-mediated pathway integration.

(**a**) Assembly of backbone plasmids to be used in cassette plasmid construction by Golden Gate method. Constructs contain two connector regions flanking a GFP expression cassette. A typical Golden Gate assembly thermocycle protocol ends on a final restriction digest step to ensure that only correctly assembled constructs are transformed. However, because these backbone plasmids contain BsaI sites by design, the assembly reactions were terminated in a final ligation step, in which the GFP expression cassette was joined between the flanking connectors. (**b**) Assembly of part plasmids into cassette plasmids. Each assembly is generated from three part plasmids―promoter, gene, and terminator―and a backbone plasmid. The backbone plasmid contains a GFP expression cassette, so that transformants containing successful assemblies can be easily distinguished from transformants containing the parent plasmid based on colony fluorescence. (**c**) Cassette plasmids are assembled into repair template plasmids. Unique restriction enzymes flank the repair template sequence, allowing for fragment linearization preceding brewer’s yeast transformation.


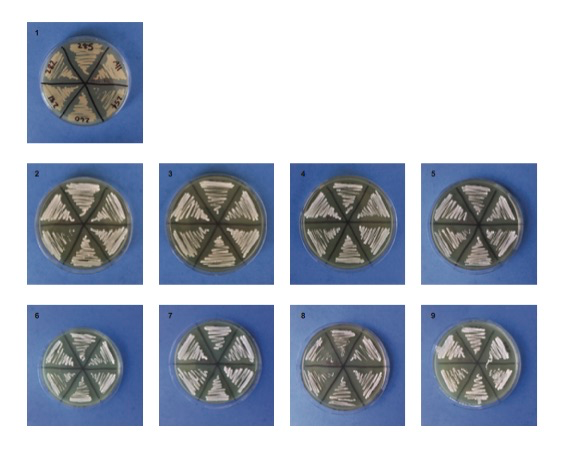


**Supplementary Figure 4** Stable engineered brewer’s yeast strains exhibited white colony phenotype upon several passages. Six strains were chosen from collection and restreaked over nine passages to confirm genetic stability.


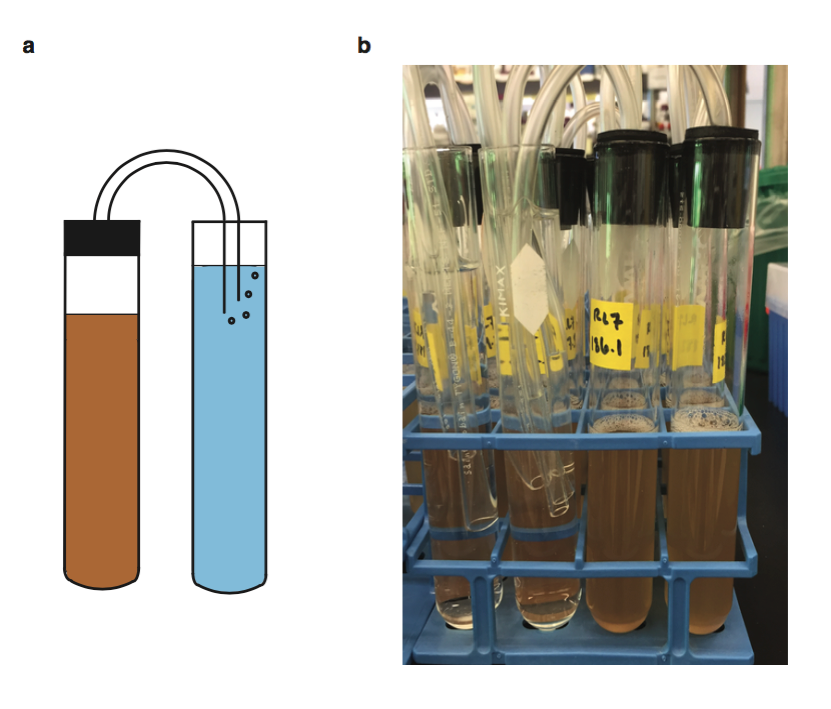


**Supplementary Figure 5** Brewer’s yeast grown in microaerobic fermentation conditions.

(**a**) Schematic diagram and (**b**) picture illustrating microaerobic fermentation conditions. Culture vials were equipped with a one-way airlock to mimic industrial brewing conditions: the airlock allows CO_2_ to evolve during fermentation while preventing oxygen uptake.


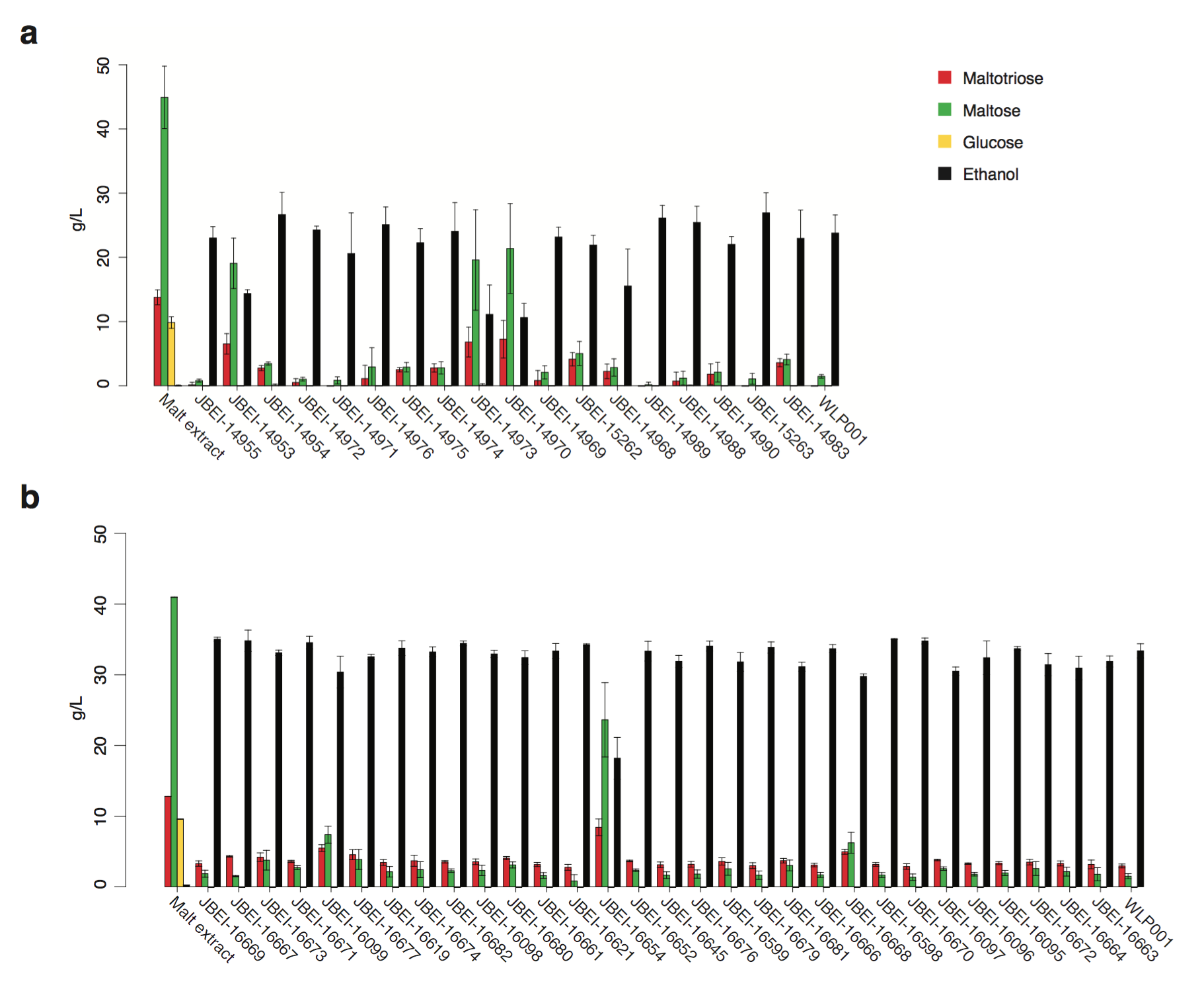


**Supplementary Figure 6** Fermentable sugars and ethanol remaining/produced after fermentation by engineered brewing strains from first (**a**) and second iteration strains (**b**) compared to parent strain *WLP001* and unfermented medium. Error bars correspond to mean ± standard deviation of three biological replicates.


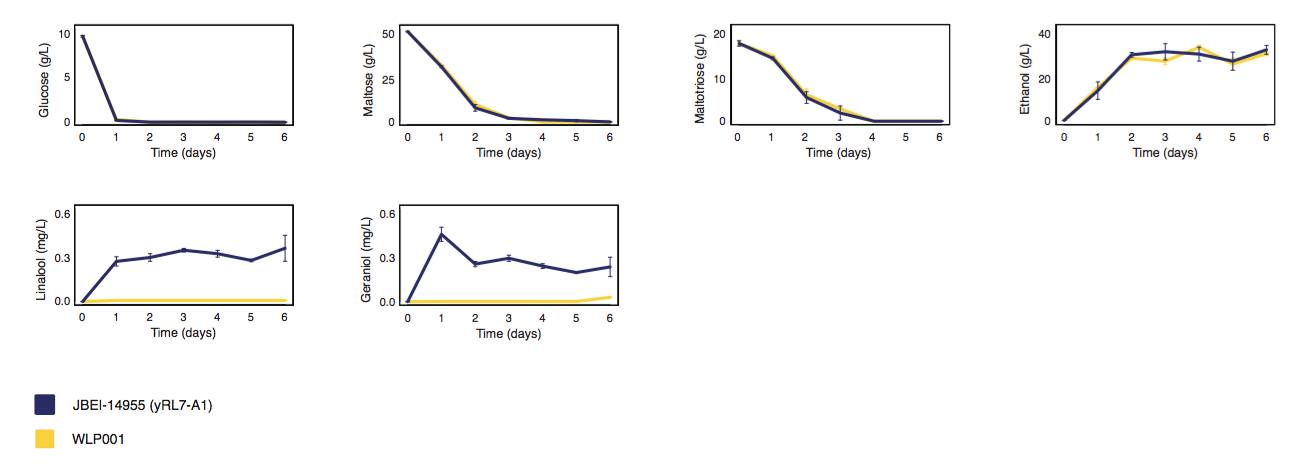


**Supplementary Figure 7** Fermentable sugars, ethanol, and monoterpene concentrations over the course of a 6-day fermentation. Engineered strain exhibits indistinguishable rates of sugar consumption and ethanol production. Error bars represent the standard deviation or 3 biological replicates.


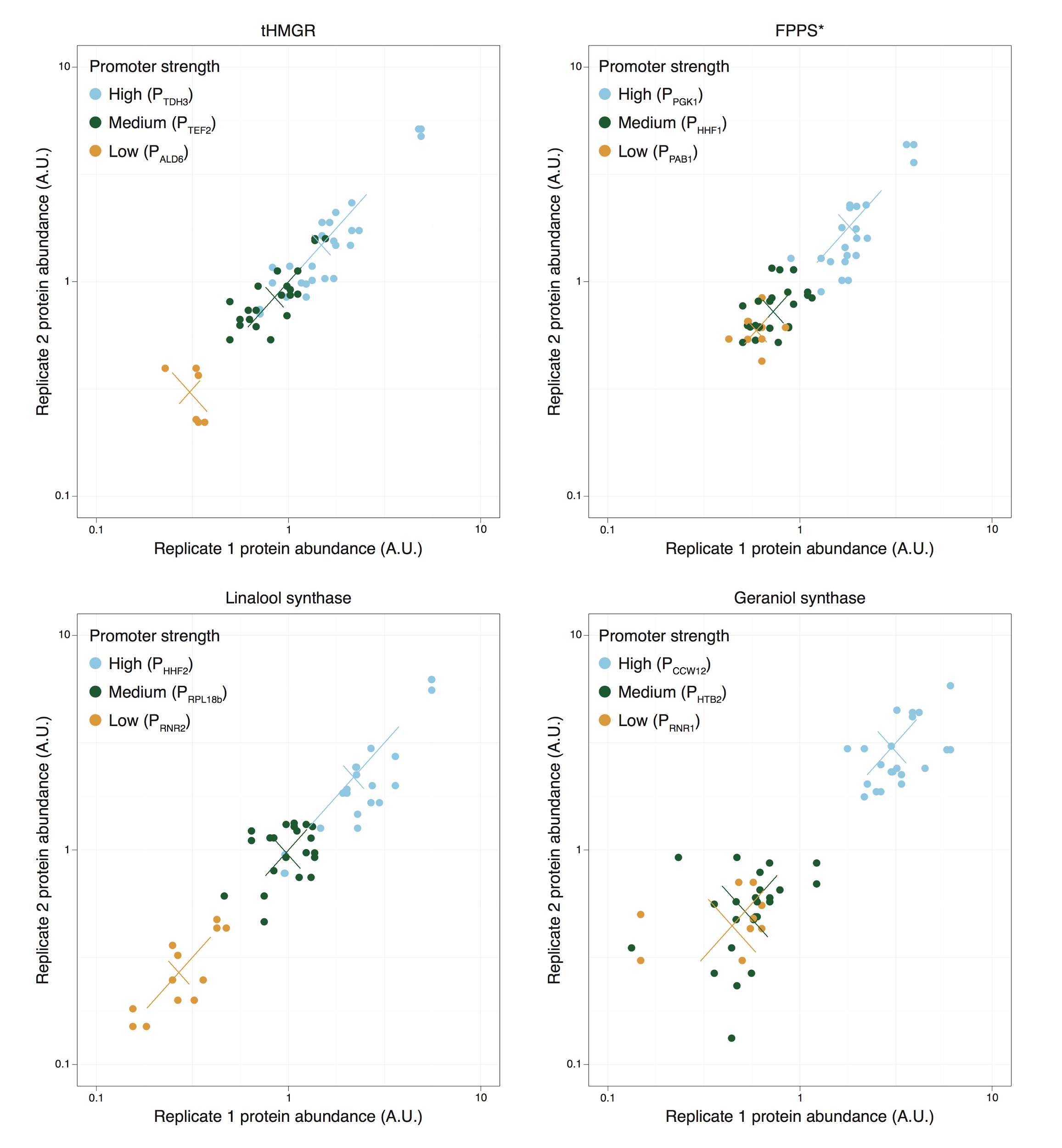


**Supplementary Figure 8** Sources of variation for protein abundance data from initial test set.

Pairwise comparison of protein abundance values between experimental replicates illustrates magnitude of variation due to promoter, genetic context and experiment. Each point represents protein abundance values for a pair of biological replicates. Each pairwise comparison is color-coded by promoter. Variation due to promoter identity can be visualized as the distance between groups of colored dots along the line *y=x*. Variation due to genetic context is reflected by the deviation within a group of colored dots along the line *y=x*. Experimental variation is reflected by the deviation within a group of colored dots in the distance from the line *y=x.*


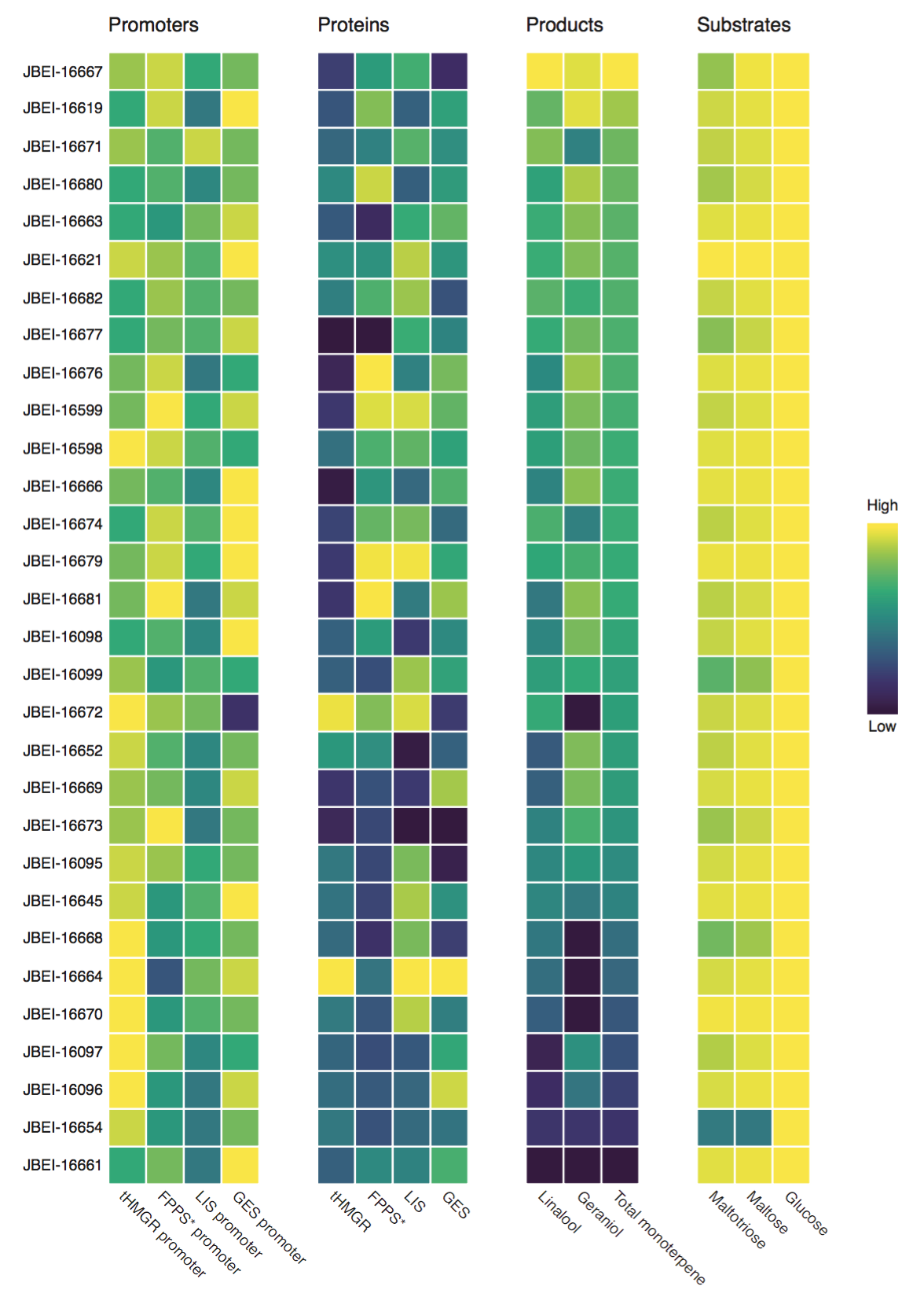


**Supplementary Figure 9** Relative promoter strengths, protein abundances, metabolites produced, and substrates consumed for the second group of brewing strains. Strains are sorted based on total monoterpene production.


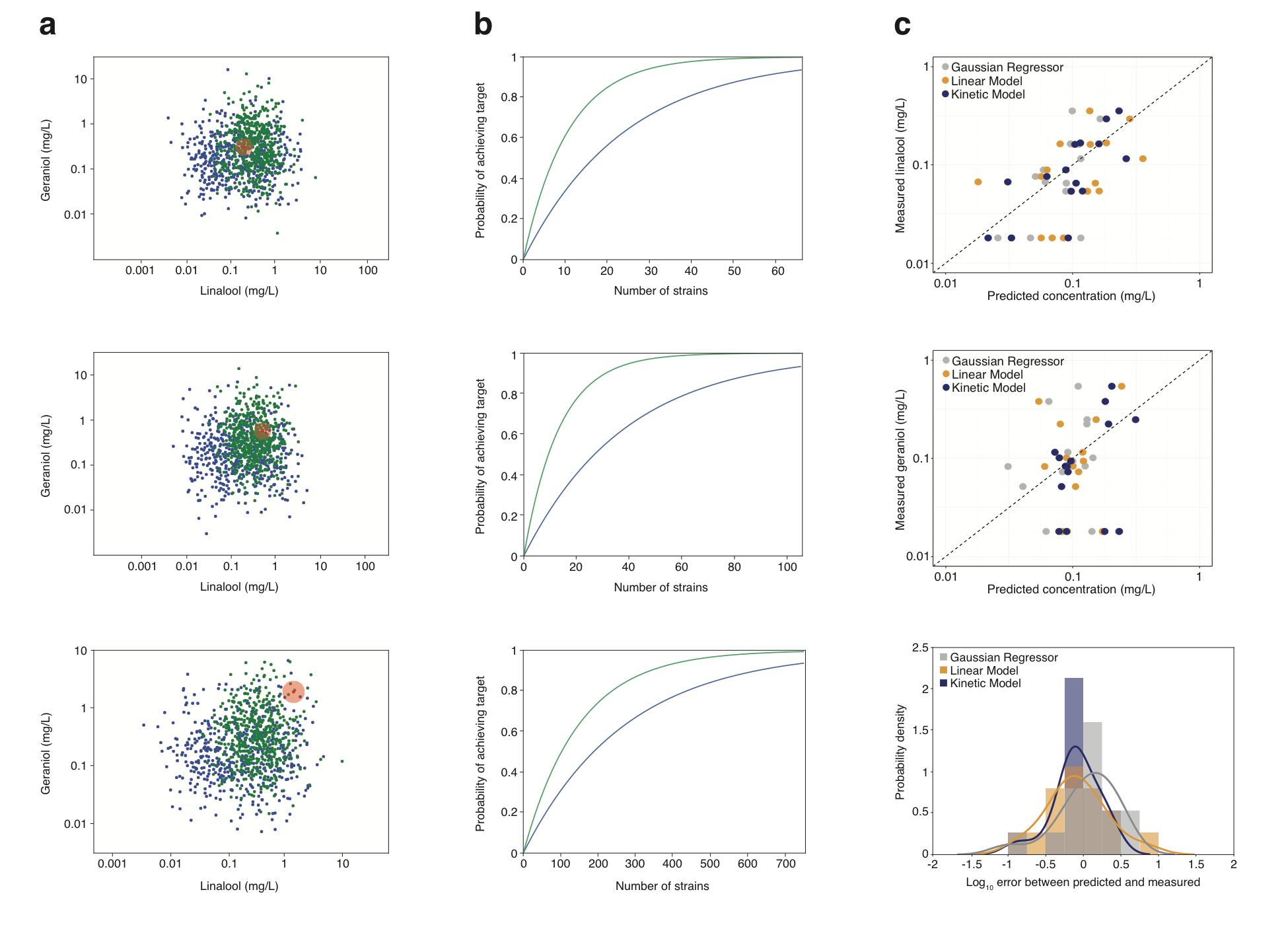


**Supplementary Figure 10** Predicting the extent of performance improvement for second iteration strains compared with randomly designed strains.

(**a**) Simulated linalool and geraniol production corresponding to guided (blue) and naive (green) genetic designs. The red point represents the target beer and the surrounding red circle represents the area within 50% of the target concentrations. (**b**) Plots represent the number of strains expected to achieve target monoterpene concentrations for sampled commercial beers. Green corresponds to second iteration strains, blue corresponds to randomly designed strains. Target beers are (top to bottom) Sierra Nevada Pale Ale, Torpedo Extra IPA, and Hop Hunter IPA. In order to achieve a 90% chance of success at meeting the target terpene concentration, the number of strains from iteration two that would need to be constructed were predicted to be 26, 32, and 356 respectively. The predicted number of strains to construct are an improvement over random selection by 55.5% on average. (**c**) Relationship between predicted and measured monoterpene concentration of first iteration strains. Three models were used to describe the relationship between protein levels and monoterpene production. The Gaussian regressor model is an interpolation scheme with no biological intuition, the linear model is a composite of two linear regression models and captures biological insights gleaned from the data, and the kinetic model is structured according to principles of Michaelis-Menten enzyme kinetics (Online Methods, Supplementary Note 3). Error residuals of the three models are shown at the bottom. Residuals that are closely centered around zero correspond to a more accurate model fit.

**Supplementary Figure 11** Sugar consumption, pH measurements, and GC/MS analysis of industrial-scale fermentations.

Fermentable sugar consumption (**a**), pH measurements (**b**), and linalool and geraniol production (**c**) during industrial fermentations. The top row corresponds to the first set of pilot fermentations, the bottom row to the second set. Sugar consumption was measured using the Plato scale (for reference, a 12 °P wort contains 12 g sugar and other soluble components per 100 g wort). Interestingly, JBEI-14971 (in the first set) was determined to be significantly different from the *WLP001* control by sensory analysis yet it behaved most similar to the parent strain during fermentation.


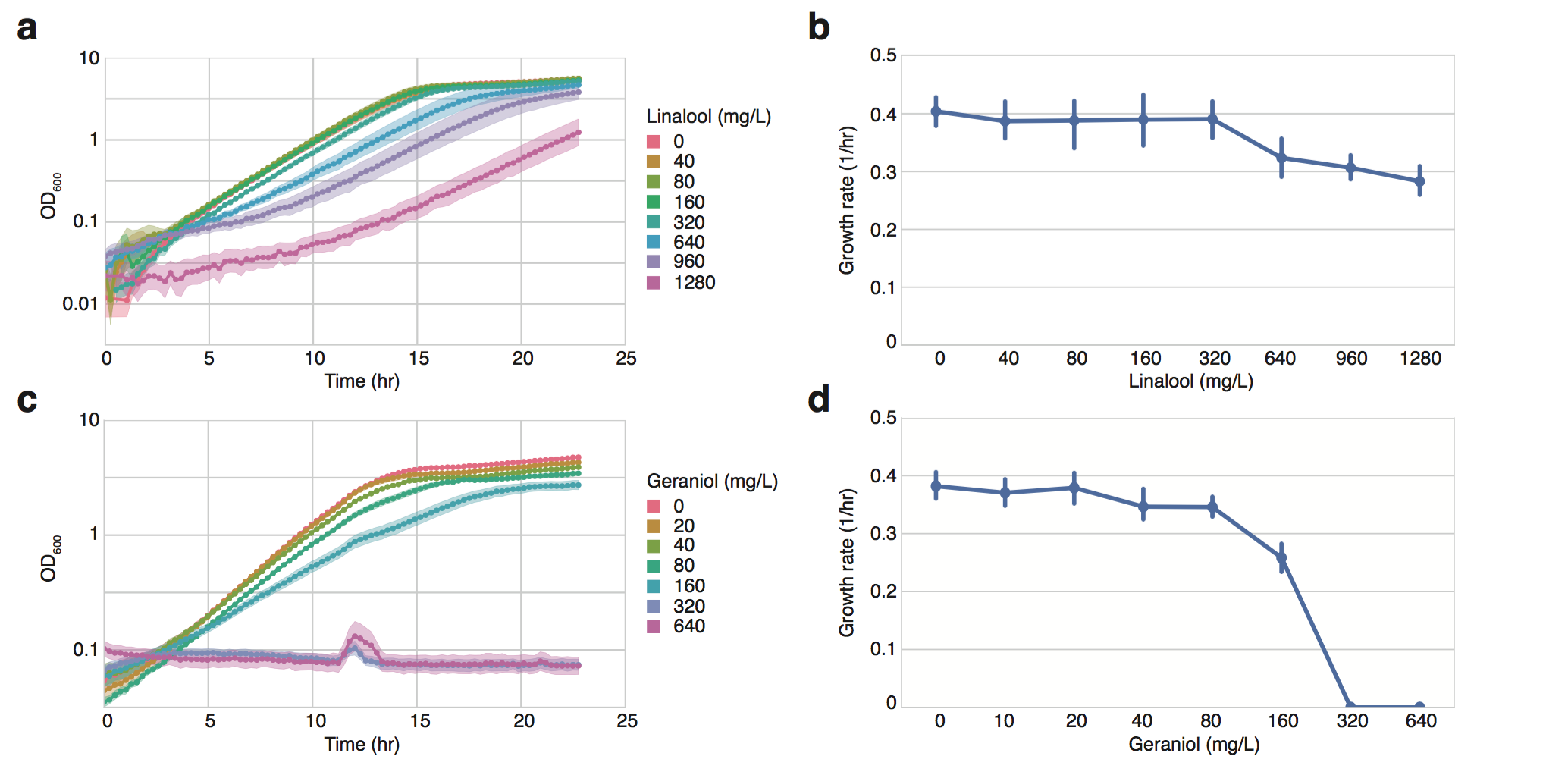


**Supplementary Figure 12** Toxicity of linalool and geraniol to brewer’s yeast strain *WLP001*.

(**a**,**c**) Growth of brewer’s yeast in varying concentrations of linalool and geraniol. (**b**,**d**) Growth rates over a range of linalool and geraniol concentrations. Statistically significant growth defects are observed in media containing 640 mg/L linalool or 160 mg/L geraniol. Growth curves were calculated by averaging 6 biological replicates; shaded areas represent one standard deviation above and below the mean. Growth rates were calculated with a sliding window of 5 hours, solving for maximum growth rate. Growth rates are presented as the average of 6 biological replicates; error bars represent 95% confidence intervals


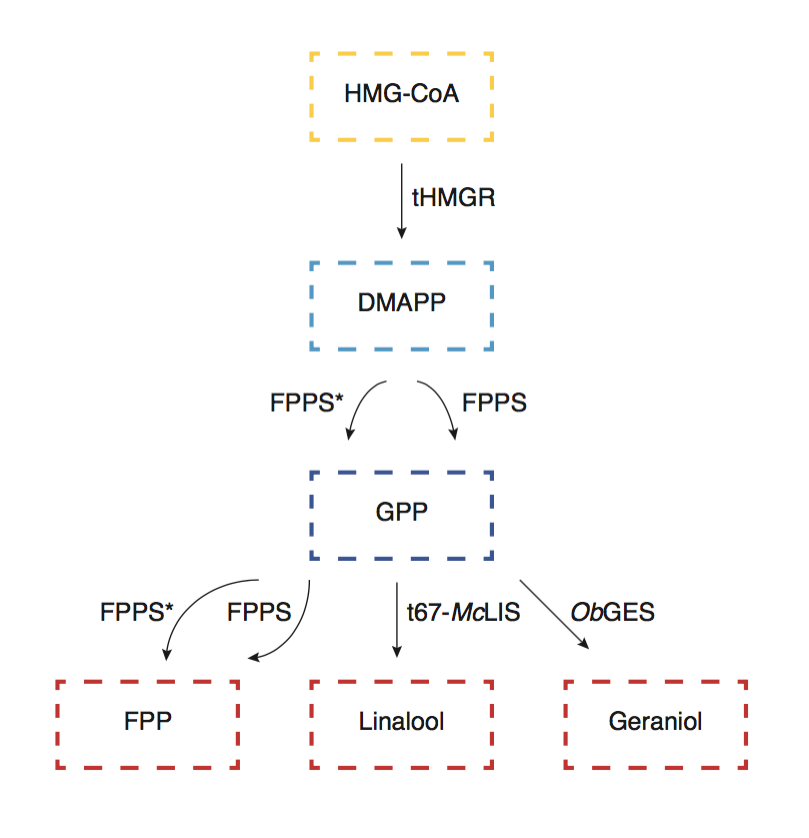


**Supplementary Figure 13** Structure of kinetic model used to describe relationship between relative protein levels and monoterpene production.

Metabolite abbreviations: HMG-CoA, 3-Hydroxy-3-MethylGlutaryl-coenzyme a; DMAPP, Dimethylallyl pyrophosphate; GPP, geranyl pyrophosphate; FPP, farnesyl pyrophosphate

Enzyme abbreviations: tHMGR, truncated HMG-CoA reductase; FPPS, farnesyl pyrophosphate synthase; *Mc*LIS, *M. citrata* linalool synthase; *Ob*GES, *O. basilicum* geraniol synthase

#

#

# **Supplementary Table 1** Correlations between strain characteristics

|  |  | tHMGR | FPPS | Linalool synthase | Geraniol synthase | Combined |
| --- | --- | --- | --- | --- | --- | --- |
| Promoter and protein | Correlation | 7.36E-01 | 8.16E-01 | 8.93E-01 | 8.66E-01 | 6.81E-01 |
|  | p-value | 5.02E-04 | 3.60E-05 | 6.24E-07 | 3.34E-06 | 4.50E-11 |
| Promoter and linalool | Correlation | 2.73E-01 | 4.93E-01 | 7.35E-01 | 2.15E-01 | 3.95E-01 |
|  | p-value | 2.74E-01 | 3.75E-02 | 5.05E-04 | 3.91E-01 | 5.94E-04 |
| Promoter and geraniol | Correlation | 5.94E-01 | 3.89E-01 | 1.81E-01 | 1.88E-01 | 2.87E-01 |
|  | p-value | 9.39E-03 | 1.10E-01 | 4.73E-01 | 4.54E-01 | 1.46E-02 |
| Promoter and total monoterpene | Correlation | 3.42E-01 | 5.83E-01 | 5.80E-01 | 2.84E-01 | 4.01E-01 |
|  | p-value | 1.65E-01 | 1.11E-02 | 1.17E-02 | 2.54E-01 | 4.75E-04 |
| Protein and linalool | Correlation | 5.12E-01 | 6.52E-01 | 8.21E-01 | 3.08E-01 | 5.35E-01 |
|  | p-value | 2.99E-02 | 3.39E-03 | 2.98E-05 | 2.14E-01 | 1.31E-06 |
| Protein and geraniol | Correlation | 6.53E-01 | 4.26E-01 | 2.76E-01 | 2.24E-01 | 3.65E-01 |
|  | p-value | 3.28E-03 | 7.76E-02 | 2.67E-01 | 3.72E-01 | 1.63E-03 |
| Protein and total monoterpene | Correlation | 5.94E-01 | 6.99E-01 | 6.72E-01 | 3.62E-01 | 5.46E-01 |
|  | p-value | 9.36E-03 | 1.26E-03 | 2.25E-03 | 1.40E-01 | 7.11E-07 |

#

#

# **Supplementary Table 2** Statistics reflecting predictivity of mathematical modeling strategies^*^

| Model type | | Mu | Sigma | Expected accuracy (90%) |
| --- | --- | --- | --- | --- |
| Linear | Linalool | 0.28 | 0.91 | 1.50 |
|  | Geraniol | 0.31 | 1.21 | 1.99 |
|  | Total | 0.20 | 0.93 | 1.53 |
| Gaussian | Linalool | 0.03 | 0.76 | 1.24 |
|  | Geraniol | 0.16 | 1.25 | 2.06 |
|  | Total | -0.02 | 0.88 | 1.45 |
| Kinetic | Linalool | 0.17 | 0.65 | 1.07 |
|  | Geraniol | 0.42 | 1.07 | 1.76 |
|  | Total | 0.22 | 0.72 | 1.18 |

^*^The moments of the error residuals are reported for each model in natural log space. Mu and sigma are the mean and standard deviation of the natural log error residuals. A zero value means zero model error. Expected accuracy is the size of the interval that captures 90% of the residual error in the model. In other words, the accuracy tells us that we expect 90% of the predictions to be within the provided window size in natural log error.

#

#

# **Supplementary Table 3** Equations used for implementing linear model^*^

| # | Equation |
| --- | --- |
| 1 | Total terpene production = α1[tHMGR] + α2[FPPS] + α3 |
| 2 | % geraniol production = α4[LIS] + α5[GES] + α6 |
| 3 | % linalool production = α7[LIS] + α8[GES] + α9 |
| 4 | Linalool = Total terpene production$\times$% linalool production |
| 5 | Geraniol = Total terpene production$\times$% geraniol production |

^*^The model was structured to reflect the observations that, 1) abundance of FPPS and tHMGR are positively correlated with monoterpene production, and 2) relative abundance of t67-*Mc*LIS, and *Ob*GES correlated with ratio of monoterpenes linalool and geraniol.

#

#

# **Supplementary Table 4** Equations used for implementing kinetic model^*^

| # | Equation |
| --- | --- |
| 1 | $\frac{d[DMAPP]}{dt}$= $\frac{Kcat1[tHMGR][HMG-CoA]}{Km1 + [HMG-CoA]}$-$\frac{Kcat2[FPPS1][DMAPP]}{Km2 + [DMAPP]}$- $\frac{Kcat4[FPPS2][DMAPP]}{Km4 + [DMAPP]}$ |
| 2 | $\frac{d[GPP]}{dt}$=$\frac{Kcat2[FPPS1][DMAPP]}{Km2 + [DMAPP]}$+ $\frac{Kcat4[FPPS2][DMAPP]}{Km4 + [DMAPP]}$- $\frac{Kcat3[FPPS1][GPP]}{Km3 + [GPP]}$-  $\frac{Kcat5[FPPS2][GPP]}{Km5 + [GPP]}$- $\frac{Kcat6[LIS][GPP]}{Km6 + [GPP]}$- $\frac{Kcat7[GES][GPP]}{Km7 + [GPP]}$ |
| 3 | $\frac{d[Linalool]}{dt}$=$\frac{Kcat6[LIS][GPP]}{Km6 + [GPP]}$ |
| 4 | $\frac{d[Geraniol]}{dt}$=$\frac{Kcat7[GES][GPP]}{Km7 + [GPP]}$ |
| 5 | [FPPS_1_] = α_0_β[FPPS] |
| 6 | [FPPS_2_] = α_0_(1 − β)[FPPS] |

^*^K_cat_s and K_m_s are numbered 1-7, see Supplementary Table 5 for corresponding enzymes. FPPS1 corresponds to the endogenous FPPS activity; FPPS2 corresponds to the FPPS* mutant activity.

#

#

# **Supplementary Table 5** Enzyme constants used in modeling studies^*^

| **Enzyme** | ***k*_cat_ (s^-1^)** | | ***K*_m_ (M)** | |
| --- | --- | --- | --- | --- |
| *S. cerevisiae* HMG-CoA reductase (ScHMGR)[^1^](https://paperpile.com/c/3CUptU/nGBhm) | *k*_cat1_ | 12926 | *K*_m1_ | 2.4×10^-6^ |
| *S. cerevisiae* FPP synthase (FPPS_1_)[^2^](https://paperpile.com/c/3CUptU/ZgFwx) | *K*_cat2 (DMAPP)_ | 0.25 | *K*_m2 (DMAPP)_ | 0.17×10^-6^ |
|  | *K*_cat3 (GPP)_ | 0.63 | *K*_m3 (GPP)_ | 0.43×10^-6^ |
| *S. cerevisiae* FPP/GPP synthase  (ScERG20* F96W-N127W, FPPS_2_)[^2^](https://paperpile.com/c/3CUptU/ZgFwx) | *K*_cat4 (DMAPP)_ | 0.012 | *K*_m4 (DMAPP)_ | 0.49×10^-6^ |
|  | *K*_cat5 (GPP)_ | 0.021 | *K*_m5 (GPP)_ | 27.56×10^-6^ |
| *M. citrata* linalool synthase (McLIS)[^3^](https://paperpile.com/c/3CUptU/cIN53) | *k*_cat6_ | 0.24 | *K*_m6_ | 25×10^-6^ |
| *O. basilicum* geraniol synthase (ObGES)[^4^](https://paperpile.com/c/3CUptU/bPm5P) | *k*_cat7_ | 0.8 | *K*_m7_ | 21×10^-6^ |

^*^Kinetic coefficients scraped from the literature served as starting points for the model parameters and were allowed to vary over an order of magnitude to improve model fit.

#

#

# **Supplementary Table 6** Parental yeast strains used in this study

| Strain | Parent strain | Description | Reference |
| --- | --- | --- | --- |
| CEN.PK2-1D |  | *MATα ura3-52; trp1-289; leu2-3,112; his3*Δ *1; MAL2-8C; SUC2* | Euroscarf |
| JBEI-14984 | CEN.PK2-1D | *leu2::*P_GAL1_-ERG8-P_GAL10_-ERG19-S.p.HIS3 | This study |
| JBEI-14985 | JBEI-14984 | *leu2::*P_GAL1_*-*ERG8-P_GAL10_-ERG19-S.p.HIS3; *ura3:*:P_GAL1_*-*ERG20-F96WN127W-P_GAL10_*-*IDI1-kanMX4 | This study |
| JBEI-14986 | JBEI-14985 | *leu2::*P_GAL1_*-*ERG8*-*P_GAL10_*-*ERG19-S.p.HIS3; *ura3:*:P_GAL1_*-*ERG20-F96WN127W-P_GAL10_*-*IDI1-kanMX4;  HMG1::HphMX4-*Ef*MvaE*-*P_GAL1/10_-*Ef*MvaS-P_ADH1_*-*trnc530-HMG1 | This study |
| WLP001 |  | California Ale Yeast | White Labs |
| JBEI-13510 | WLP001 | *ADE2*Δ | This study |

#

#

# **Supplementary Table 7** Plasmids used for construction of GPP-hyper-producing strain

| JBEI Number | Description | Marker | Ori | Related plasmid/strain |
| --- | --- | --- | --- | --- |
| JBEI-16636 | LEU2-HR-*Sk*His3MX4  P_GAL1_-ERG8-T_ERG8_  P_GAL10_-ERG19-T_ERG19_  LEU2-HR | Carb | ColE1 |  |
| JBEI-12291 | URA3-HR-KanMX4  P_GAL1_*-*ERG20-F96WN127W-T_CYC1_  P_GAL10_*-*IDI1-T_ADH1_  URA3-HR | Carb | ColE1 | JBEI-14985 |
| JBEI-12289 | HMG1-HR-HphMX4  *Ef*MvaE-T_ADH1_ | Carb | ColE1 | JBEI-14986 |
| JBEI-12285 | P_GAL1_-*Ef*MvaE(100nts)  P_GAL1_-*Ef*MvaS-T_CYC1_  P_ADH1_-trnc530-HMG1 | Carb | ColE1 | JBEI-14986 |

#

# **Supplementary Table 8** Plasmids used for heterologous protein expression in yeast^*^

| JBEI Number | Description | Marker | Ori | Related Figure |
| --- | --- | --- | --- | --- |
| JBEI-10738 | pESC-LEU2 (Invitrogen) | LEU2 | 2μ | Fig. 1c |
| JBEI-13024 | pEntry_P_GAL1_-sfGFP  (pEntry_GoldenGate) | LEU2 | 2μ | Fig. 1c |
| JBEI-12279 | P_GAL1_-*Cb*LIS | LEU2 | 2μ | Fig. 1c |
| JBEI-13019 | P_GAL1_-*Cb*LIS-native | LEU2 | 2μ | Fig. 1c |
| JBEI-12313 | P_GAL1_-*Ap*LIS | LEU2 | 2μ | Fig. 1c |
| JBEI-12298 | P_GAL1_-*Fa*LIS | LEU2 | 2μ | Fig. 1c |
| JBEI-12304 | P_GAL1_-*Pf*LIS | LEU2 | 2μ | Fig. 1c |
| JBEI-13022 | P_GAL1_-*Mc*LIS | LEU2 | 2μ | Fig. 1c |
| JBEI-12334 | P_GAL1_-*Le*LIS | LEU2 | 2μ | Fig. 1c |
| JBEI-12299 | P_GAL1_-trnc26-*Ap*LIS | LEU2 | 2μ | Fig. 1c |
| JBEI-12303 | P_GAL1_-trnc34-*Pf*LIS | LEU2 | 2μ | Fig. 1c |
| JBEI-13018 | P_GAL1_-trnc51-*Mc*LIS | LEU2 | 2μ | Fig. 1c |
| JBEI-12314 | P_GAL1_-trnc43-*Le*LIS | LEU2 | 2μ | Fig. 1c |
| JBEI-12306 | P_GAL1_-trnc19-*Fa*LIS | LEU2 | 2μ | Fig. 1c |
| JBEI-12332 | P_GAL1_-trnc60-*Pf*LIS | LEU2 | 2μ | Fig. 1c |
| JBEI-13017 | P_GAL1_-trnc67-*Mc*LIS | LEU2 | 2μ | Fig. 1c |
| JBEI-14916 | P_GAL1_-*Cj*GES | LEU2 | 2μ | Fig. 1d |
| JBEI-14921 | P_GAL1_-*Oe*GES | LEU2 | 2μ | Fig. 1d |
| JBEI-14922 | P_GAL1_-*Pf*GES | LEU2 | 2μ | Fig. 1d |
| JBEI-14923 | P_GAL1_-*Pd*GES | LEU2 | 2μ | Fig. 1d |
| JBEI-14924 | P_GAL1_-*Vv*GES | LEU2 | 2μ | Fig. 1d |
| JBEI-14917 | P_GAL1_-*Ob*GES | LEU2 | 2μ | Fig. 1d |
| JBEI-14918 | P_GAL1_-trnc30-*Cj*GES | LEU2 | 2μ | Fig. 1d |
| JBEI-14919 | P_GAL1_-trnc58-*Oe*GES | LEU2 | 2μ | Fig. 1d |
| JBEI-14920 | P_GAL1_-trnc35-*Pf*GES | LEU2 | 2μ | Fig. 1d |
| JBEI-14931 | P_GAL1_-trnc43-*Pd*GES | LEU2 | 2μ | Fig. 1d |
| JBEI-14929 | P_GAL1_-trnc71-*Vv*GES | LEU2 | 2μ | Fig. 1d |
| JBEI-14930 | P_GAL1_-trnc63-*Ob*GES | LEU2 | 2μ | Fig. 1d |
| JBEI-14938 | P_GAL1_-trnc52-*Cj*GES | LEU2 | 2μ | Fig. 1d |
| JBEI-14936 | P_GAL1_-trnc91-*Oe*GES | LEU2 | 2μ | Fig. 1d |
| JBEI-14942 | P_GAL1_-trnc59-*Pf*GES | LEU2 | 2μ | Fig. 1d |
| JBEI-14940 | P_GAL1_-trnc86-*Pd*GES | LEU2 | 2μ | Fig. 1d |
| JBEI-14946 | P_GAL1_-trnc78-*Vv*GES | LEU2 | 2μ | Fig. 1d |
| JBEI-14943 | P_GAL1_-trnc72-*Ob*GES | LEU2 | 2μ | Fig. 1d |

^*^Species of origin: *Clarkia brewerii*[*^5^*](https://paperpile.com/c/3CUptU/Zq9MN), *Actinidia polygama*[*^6^*](https://paperpile.com/c/3CUptU/EV6Sh), *Fragaria x ananassa*[*^7^*](https://paperpile.com/c/3CUptU/NByyB), *Perilla frutescens*[*^8,9^*](https://paperpile.com/c/3CUptU/3tfSg+suUyy), *Mentha citrata*[*^10^*](https://paperpile.com/c/3CUptU/rXp40), *Lycopersicon esculentum*[*^11^*](https://paperpile.com/c/3CUptU/U903r), *Citrus jambhiri*[*^12^*](https://paperpile.com/c/3CUptU/ojYfJ), *Olea europaea*[*^13^*](https://paperpile.com/c/3CUptU/hj87I), *Phyla dulcis*[*^14^*](https://paperpile.com/c/3CUptU/kgk9Z), *Vitis vinifera*[*^15^*](https://paperpile.com/c/3CUptU/e1WPU), *Ocimum basilicum*[*^16^*](https://paperpile.com/c/3CUptU/klNCw)

# **Supplementary Table 9** Part plasmids generated in this study

| JBEI Number | Description | Marker | Ori | Related Figure |
| --- | --- | --- | --- | --- |
| JBEI-13815 | trnc530-HMG1 | Cm | ColE1 | Fig. 2d, Fig. S3 |
| JBEI-13814 | ERG20-F96WN127W | Cm | ColE1 | Fig. 2d, Fig. S3 |
| JBEI-13550 | trnc67-*Mc*LIS | Cm | ColE1 | Fig. 2d, Fig. S3 |
| JBEI-13546 | *Ob*GES | Cm | ColE1 | Fig. 2d, Fig. S3 |

#

# **Supplementary Table 10** Pathway plasmids used for construction of initial test set of industrial brewing strains

| JBEI Number | Description | Marker | Ori | Related Figure |
| --- | --- | --- | --- | --- |
| JBEI-13898 | pEntry-ADE2 | KanR | ColE1 | Fig. 2d, Fig. S3 |
| JBEI-14122 | P_TDH3_-trnc530-HMG1-T_ENO1_  P_HHF1_-ERG20-F96WN127W-T_SSA1_  P_RPL18b_-trnc67-*Mc*LIS-T_ADH1_  P_HTB2_-*Ob*GES-T_PGK1_ | KanR | ColE1 | Fig. 2d, Fig. S3 |
| JBEI-14421 | P_ALD6_-trnc530-HMG1-T_ENO1_  P_PGK1_-ERG20-F96WN127W-T_SSA1_  P_HHF2_-trnc67-*Mc*LIS-T_ADH1_  P_CCW12_-*Ob*GES-T_PGK1_ | KanR | ColE1 | Fig. 2d, Fig. S3 |
| JBEI-14423 | P_TDH3_-trnc530-HMG1-T_ENO1_  P_PGK1_-ERG20-F96WN127W-T_SSA1_  P_RPL18b_-trnc67-*Mc*LIS-T_ADH1_  P_CCW12_-*Ob*GES-T_PGK1_ | KanR | ColE1 | Fig. 2d, Fig. S3 |
| JBEI-14424 | P_TDH3_-trnc530-HMG1-T_ENO1_  P_PGK1_-ERG20-F96WN127W-T_SSA1_  P_HHF2_-trnc67-*Mc*LIS-T_ADH1_  P_CCW12_-*Ob*GES-T_PGK1_ | KanR | ColE1 | Fig. 2d, Fig. S3 |
| JBEI-14438 | P_TEF2_-trnc530-HMG1-T_ENO1_  P_PGK1_-ERG20-F96WN127W-T_SSA1_  P_HHF2_-trnc67-*Mc*LIS-T_ADH1_  P_CCW12_-*Ob*GES-T_PGK1_ | KanR | ColE1 | Fig. 2d, Fig. S3 |
| JBEI-14458 | P_TDH3_-trnc530-HMG1-T_ENO1_  P_PGK1_-ERG20-F96WN127W-T_SSA1_  P_HHF2_-trnc67-*Mc*LIS-T_ADH1_  P_HTB2_-*Ob*GES-T_PGK1_ | KanR | ColE1 | Fig. 2d, Fig. S3 |
| JBEI-14460 | P_TDH3_-trnc530-HMG1-T_ENO1_  P_HHF1_-ERG20-F96WN127W-T_SSA1_  P_HHF2_-trnc67-*Mc*LIS-T_ADH1_  P_CCW12_-*Ob*GES-T_PGK1_ | KanR | ColE1 | Fig. 2d, Fig. S3 |
| JBEI-14925 | P_TEF2_-trnc530-HMG1-T_ENO1_  P_PGK1_-ERG20-F96WN127W-T_SSA1_  P_RPL18b_-trnc67-*Mc*LIS-T_ADH1_  P_RNR1_-*Ob*GES-T_PGK1_ | KanR | ColE1 | Fig. 2d, Fig. S3 |
| JBEI-14926 | P_TEF2_-trnc530-HMG1-T_ENO1_  P_PGK1_-ERG20-F96WN127W-T_SSA1_  P_HHF2_-trnc67-*Mc*LIS-T_ADH1_  P_RNR1_-*Ob*GES-T_PGK1_ | KanR | ColE1 | Fig. 2d, Fig. S3 |
| JBEI-14928 | P_TDH3_-trnc530-HMG1-T_ENO1_  P_PAB1_-ERG20-F96WN127W-T_SSA1_  P_RNR2_-trnc67-*Mc*LIS-T_ADH1_  P_HTB2_-*Ob*GES-T_PGK1_ | KanR | ColE1 | Fig. 2d, Fig. S3 |
| JBEI-14932 | P_TDH3_-trnc530-HMG1-T_ENO1_  P_PAB1_-ERG20-F96WN127W-T_SSA1_  P_RPL18b_-trnc67-*Mc*LIS-T_ADH1_  P_HTB2_-*Ob*GES-T_PGK1_ | KanR | ColE1 | Fig. 2d, Fig. S3 |
| JBEI-14935 | P_TEF2_-trnc530-HMG1-T_ENO1_  P_PAB1_-ERG20-F96WN127W-T_SSA1_  P_RPL18b_-trnc67-*Mc*LIS-T_ADH1_  P_HTB2_-*Ob*GES-T_PGK1_ | KanR | ColE1 | Fig. 2d, Fig. S3 |
| JBEI-14937 | P_ALD6_-trnc530-HMG1-T_ENO1_  P_HHF1_-ERG20-F96WN127W-T_SSA1_  P_RPL18b_-trnc67-*Mc*LIS-T_ADH1_  P_CCW12_-*Ob*GES-T_PGK1_ | KanR | ColE1 | Fig. 2d, Fig. S3 |
| JBEI-14945 | P_TEF2_-trnc530-HMG1-T_ENO1_  P_HHF1_-ERG20-F96WN127W-T_SSA1_  P_RNR2_-trnc67-*Mc*LIS-T_ADH1_  P_CCW12_-*Ob*GES-T_PGK1_ | KanR | ColE1 | Fig. 2d, Fig. S3 |
| JBEI-14948 | P_TEF2_-trnc530-HMG1-T_ENO1_  P_HHF1_-ERG20-F96WN127W-T_SSA1_  P_RNR2_-trnc67-*Mc*LIS-T_ADH1_  P_HTB2_-*Ob*GES-T_PGK1_ | KanR | ColE1 | Fig. 2d, Fig. S3 |
| JBEI-14949 | P_TDH3_-trnc530-HMG1-T_ENO1_  P_HHF1_-ERG20-F96WN127W-T_SSA1_  P_RPL18b_-trnc67-*Mc*LIS-T_ADH1_  P_RNR1_-*Ob*GES-T_PGK1_ | KanR | ColE1 | Fig. 2d, Fig. S3 |
| JBEI-14950 | P_TEF2_-trnc530-HMG1-T_ENO1_  P_HHF1_-ERG20-F96WN127W-T_SSA1_  P_RNR2_-trnc67-*Mc*LIS-T_ADH1_  P_RNR1_-*Ob*GES-T_PGK1_ | KanR | ColE1 | Fig. 2d, Fig. S3 |

# **Supplementary Table 11** Pathway plasmids used for construction of second set of industrial brewing strains

| JBEI Number | Description | Marker | Ori | Related Figure |
| --- | --- | --- | --- | --- |
| JBEI-13898 | pENTRY-ADE2 | KanR | ColE1 | Fig. S3 |
| JBEI-16632 | P_PGK1_-trnc530-HMG1-T_ENO1_  P_TDH3_-ERG20-F96WN127W-T_SSA1_  P_RPL18b_-trnc67-*Mc*LIS-T_ADH1_  P_HHF2_-*Ob*GES-T_PGK1_ | KanR | ColE1 | Fig. S3 |
| JBEI-16633 | P_PGK1_-trnc530-HMG1-T_ENO1_  P_CCW12_-ERG20-F96WN127W-T_SSA1_  P_TEF2_-trnc67-*Mc*LIS-T_ADH1_  P_HHF2_-*Ob*GES-T_PGK1_ | KanR | ColE1 | Fig. S3 |
| JBEI-16634 | P_HHF2_-trnc530-HMG1-T_ENO1_  P_CCW12_-ERG20-F96WN127W-T_SSA1_  P_TEF2_-trnc67-*Mc*LIS-T_ADH1_  P_TDH3_-*Ob*GES-T_PGK1_ | KanR | ColE1 | Fig. S3 |
| JBEI-16635 | P_PGK1_-trnc530-HMG1-T_ENO1_  P_HHF2_-ERG20-F96WN127W-T_SSA1_  P_HTB2_-trnc67-*Mc*LIS-T_ADH1_  P_CCW12_-*Ob*GES-T_PGK1_ | KanR | ColE1 | Fig. S3 |
| JBEI-16637 | P_TDH3_-trnc530-HMG1-T_ENO1_  P_HHF1_-ERG20-F96WN127W-T_SSA1_  P_TEF1_-trnc67-*Mc*LIS-T_ADH1_  P_HHF2_-*Ob*GES-T_PGK1_ | KanR | ColE1 | Fig. S3 |
| JBEI-16638 | P_TDH3_-trnc530-HMG1-T_ENO1_  P_HHF2_-ERG20-F96WN127W-T_SSA1_  P_HTB2_-trnc67-*Mc*LIS-T_ADH1_  P_TEF2_-*Ob*GES-T_PGK1_ | KanR | ColE1 | Fig. S3 |
| JBEI-16639 | P_TDH3_-trnc530-HMG1-T_ENO1_  P_HHF1_-ERG20-F96WN127W-T_SSA1_  P_RPL18b_-trnc67-*Mc*LIS-T_ADH1_  P_CCW12_-*Ob*GES-T_PGK1_ | KanR | ColE1 | Fig. S3 |
| JBEI-16640 | P_CCW12_-trnc530-HMG1-T_ENO1_  P_PGK1_-ERG20-F96WN127W-T_SSA1_  P_TEF2_-trnc67-*Mc*LIS-T_ADH1_  P_HHF2_-*Ob*GES-T_PGK1_ | KanR | ColE1 | Fig. S3 |
| JBEI-16641 | P_HHF2_-trnc530-HMG1-T_ENO1_  P_TDH3_-ERG20-F96WN127W-T_SSA1_  P_RPL18b_-trnc67-*Mc*LIS-T_ADH1_  P_CCW12_-*Ob*GES-T_PGK1_ | KanR | ColE1 | Fig. S3 |
| JBEI-16642 | P_HHF2_-trnc530-HMG1-T_ENO1_  P_TEF1_-ERG20-F96WN127W-T_SSA1_  P_HTB2_-trnc67-*Mc*LIS-T_ADH1_  P_TDH3_-*Ob*GES-T_PGK1_ | KanR | ColE1 | Fig. S3 |
| JBEI-16643 | P_TDH3_-trnc530-HMG1-T_ENO1_  P_HHF1_-ERG20-F96WN127W-T_SSA1_  P_TEF2_-trnc67-*Mc*LIS-T_ADH1_  P_HHF2_-*Ob*GES-T_PGK1_ | KanR | ColE1 | Fig. S3 |
| JBEI-16644 | P_TDH3_-trnc530-HMG1-T_ENO1_  P_CCW12_-ERG20-F96WN127W-T_SSA1_  P_TEF1_-trnc67-*Mc*LIS-T_ADH1_  P_TEF2_-*Ob*GES-T_PGK1_ | KanR | ColE1 | Fig. S3 |
| JBEI-16646 | P_TEF2_-trnc530-HMG1-T_ENO1_  P_CCW12_-ERG20-F96WN127W-T_SSA1_  P_TEF1_-trnc67-*Mc*LIS-T_ADH1_  P_TDH3_-*Ob*GES-T_PGK1_ | KanR | ColE1 | Fig. S3 |
| JBEI-16647 | P_TEF2_-trnc530-HMG1-T_ENO1_  P_CCW12_-ERG20-F96WN127W-T_SSA1_  P_RPL18b_-trnc67-*Mc*LIS-T_ADH1_  P_TDH3_-*Ob*GES-T_PGK1_ | KanR | ColE1 | Fig. S3 |
| JBEI-16648 | P_TEF2_-trnc530-HMG1-T_ENO1_  P_HHF2_-ERG20-F96WN127W-T_SSA1_  P_RPL18b_-trnc67-*Mc*LIS-T_ADH1_  P_TDH3_-*Ob*GES-T_PGK1_ | KanR | ColE1 | Fig. S3 |
| JBEI-16649 | P_TEF2_-trnc530-HMG1-T_ENO1_  P_TEF1_-ERG20-F96WN127W-T_SSA1_  P_HTB2_-trnc67-*Mc*LIS-T_ADH1_  P_HHF2_-*Ob*GES-T_PGK1_ | KanR | ColE1 | Fig. S3 |
| JBEI-16650 | P_TEF2_-trnc530-HMG1-T_ENO1_  P_TEF1_-ERG20-F96WN127W-T_SSA1_  P_HTB2_-trnc67-*Mc*LIS-T_ADH1_  P_TDH3_-*Ob*GES-T_PGK1_ | KanR | ColE1 | Fig. S3 |
| JBEI-16651 | P_TEF2_-trnc530-HMG1-T_ENO1_  P_PGK1_-ERG20-F96WN127W-T_SSA1_  P_TEF1_-trnc67-*Mc*LIS-T_ADH1_  P_HHF2_-*Ob*GES-T_PGK1_ | KanR | ColE1 | Fig. S3 |
| JBEI-16653 | P_CCW12_-trnc530-HMG1-T_ENO1_  P_TEF1_-ERG20-F96WN127W-T_SSA1_  P_HTB2_-trnc67-*Mc*LIS-T_ADH1_  P_HHF2_-*Ob*GES-T_PGK1_ | KanR | ColE1 | Fig. S3 |
| JBEI-16655 | P_CCW12_-trnc530-HMG1-T_ENO1_  P_HHF1_-ERG20-F96WN127W-T_SSA1_  P_RPL18b_-trnc67-*Mc*LIS-T_ADH1_  P_HHF2_-*Ob*GES-T_PGK1_ | KanR | ColE1 | Fig. S3 |
| JBEI-16656 | P_CCW12_-trnc530-HMG1-T_ENO1_  P_PGK1_-ERG20-F96WN127W-T_SSA1_  P_TEF1_-trnc67-*Mc*LIS-T_ADH1_  P_TDH3_-*Ob*GES-T_PGK1_ | KanR | ColE1 | Fig. S3 |
| JBEI-16657 | P_TDH3_-trnc530-HMG1-T_ENO1_  P_PAB1_-ERG20-F96WN127W-T_SSA1_  P_HHF2_-trnc67-*Mc*LIS-T_ADH1_  P_CCW12_-*Ob*GES-T_PGK1_ | KanR | ColE1 | Fig. S3 |
| JBEI-16658 | P_TEF2_-trnc530-HMG1-T_ENO1_  P_HHF1_-ERG20-F96WN127W-T_SSA1_  P_HHF2_-trnc67-*Mc*LIS-T_ADH1_  P_CCW12_-*Ob*GES-T_PGK1_ | KanR | ColE1 | Fig. S3 |
| JBEI-16659 | P_TDH3_-trnc530-HMG1-T_ENO1_  P_PGK1_-ERG20-F96WN127W-T_SSA1_  P_HHF2_-trnc67-*Mc*LIS-T_ADH1_  P_RNR1_-*Ob*GES-T_PGK1_ | KanR | ColE1 | Fig. S3 |
| JBEI-16660 | P_TEF2_-trnc530-HMG1-T_ENO1_  P_HHF2_-ERG20-F96WN127W-T_SSA1_  P_TEF1_-trnc67-*Mc*LIS-T_ADH1_  P_CCW12_-*Ob*GES-T_PGK1_ | KanR | ColE1 | Fig. S3 |
| JBEI-16662 | P_PGK1_-trnc530-HMG1-T_ENO1_  P_HHF1_-ERG20-F96WN127W-T_SSA1_  P_TEF1_-trnc67-*Mc*LIS-T_ADH1_  P_TEF2_-*Ob*GES-T_PGK1_ | KanR | ColE1 | Fig. S3 |
| JBEI-16665 | P_PGK1_-trnc530-HMG1-T_ENO1_  P_TEF1_-ERG20-F96WN127W-T_SSA1_  P_CCW12_-trnc67-*Mc*LIS-T_ADH1_  P_HHF2_-*Ob*GES-T_PGK1_ | KanR | ColE1 | Fig. S3 |
| JBEI-16675 | P_HHF2_-trnc530-HMG1-T_ENO1_  P_CCW12_-ERG20-F96WN127W-T_SSA1_  P_RPL18b_-trnc67-*Mc*LIS-T_ADH1_  P_TEF2_-*Ob*GES-T_PGK1_ | KanR | ColE1 | Fig. S3 |
| JBEI-16678 | P_CCW12_-trnc530-HMG1-T_ENO1_  P_HHF1_-ERG20-F96WN127W-T_SSA1_  P_TEF1_-trnc67-*Mc*LIS-T_ADH1_  P_TDH3_-*Ob*GES-T_PGK1_ | KanR | ColE1 | Fig. S3 |
| JBEI-16683 | P_HHF2_-trnc530-HMG1-T_ENO1_  P_TDH3_-ERG20-F96WN127W-T_SSA1_  P_TEF2_-trnc67-*Mc*LIS-T_ADH1_  P_CCW12_-*Ob*GES-T_PGK1_ | KanR | ColE1 | Fig. S3 |

#

# **Supplementary Table 12** Strain names and genotypes of first iteration strains

| Strain | Parent strain | Description | Reference |
| --- | --- | --- | --- |
| JBEI-14953 | JBEI-13510 | *ade2*::P_ADE2_-ADE2-T_ADE2_  P_TDH3_-trnc530-HMG1-T_ENO1_  P_PGK1_-ERG20-F96WN127W-T_SSA1_  P_HHF2_-trnc67-*Mc*LIS-T_ADH1_  P_HTB2_-*Ob*GES-T_PGK1_ | This study |
| JBEI-14954 | JBEI-13510 | *ade2*::P_ADE2_-ADE2-T_ADE2_  P_TDH3_-tHMG1-T_ENO1_  P_PGK1_-ERG20-F96WN127W-T_SSA1_  P_RPL18b_-trnc67-*Mc*LIS-T_ADH1_  P_CCW12_-*Ob*GES-T_PGK1_ | This study |
| JBEI-14955 | JBEI-13510 | *ade2*::P_ADE2_-ADE2-T_ADE2_  P_TDH3_-trnc530-HMG1-T_ENO1_  P_PGK1_-ERG20-F96WN127W-T_SSA1_  P_HHF2_-trnc67-*Mc*LIS-T_ADH1_  P_CCW12_-*Ob*GES-T_PGK1_ | This study |
| JBEI-14968 | JBEI-13510 | *ade2*::P_ADE2_-ADE2-T_ADE2_  P_TEF2_-tHMG1-T_ENO1_  P_HHF1_-ERG20-F96WN127W-T_SSA1_  P_RNR2_-t67-*Mc*LIS-T_ADH1_  P_CCW12_-*Ob*GES-T_PGK1_ | This study |
| JBEI-14969 | JBEI-13510 | *ade2*::P_ADE2_-ADE2-T_ADE2_  P_TEF2_-tHMG1-T_ENO1_  P_PGK1_-ERG20-F96WN127W-T_SSA1_  P_HHF2_-t67-*Mc*LIS-T_ADH1_  P_RNR1_-*Ob*GES-T_PGK1_ | This study |
| JBEI-14970 | JBEI-13510 | *ade2*::P_ADE2_-ADE2-T_ADE2_  P_TEF2_-tHMG1-T_ENO1_  P_PGK1_-ERG20-F96WN127W-T_SSA1_  P_HHF2_-t67-*Mc*LIS-T_ADH1_  P_CCW12_-*Ob*GES-T_PGK1_ | This study |
| JBEI-14971 | JBEI-13510 | *ade2*::P_ADE2_-ADE2-T_ADE2_  P_TDH3_-tHMG1-T_ENO1_  P_HHF1_-ERG20-F96WN127W-T_SSA1_  P_HHF2_-t67-*Mc*LIS-T_ADH1_  P_HTB2_-*Ob*GES-T_PGK1_ | This study |
| JBEI-14972 | JBEI-13510 | *ade2*::P_ADE2_-ADE2-T_ADE2_  P_TDH3_-tHMG1-T_ENO1_  P_HHF1_-ERG20-F96WN127W-T_SSA1_  P_HHF2_-t67-*Mc*LIS-T_ADH1_  P_CCW12_-*Ob*GES-T_PGK1_ | This study |
| JBEI-14973 | JBEI-13510 | *ade2*::P_ADE2_-ADE2-T_ADE2_  P_TDH3_-tHMG1-T_ENO1_  P_PAB1_-ERG20-F96WN127W-T_SSA1_  P_RNR2_-t67-*Mc*LIS-T_ADH1_  P_HTB2_-*Ob*GES-T_PGK1_ | This study |
| JBEI-14974 | JBEI-13510 | *ade2*::P_ADE2_-ADE2-T_ADE2_  P_TDH3_-tHMG1-T_ENO1_  P_PAB1_-ERG20-F96WN127W-T_SSA1_  P_RPL18b_-t67-*Mc*LIS-T_ADH1_  P_HTB2_-*Ob*GES-T_PGK1_ | This study |
| JBEI-14975 | JBEI-13510 | *ade2*::P_ADE2_-ADE2-T_ADE2_  P_TDH3_-tHMG1-T_ENO1_  P_HHF1_-ERG20-F96WN127W-T_SSA1_  P_RPL18b_-t67-*Mc*LIS-T_ADH1_  P_RNR1_-*Ob*GES-T_PGK1_ | This study |
| JBEI-14976 | JBEI-13510 | *ade2*::P_ADE2_-ADE2-T_ADE2_  P_TDH3_-tHMG1-T_ENO1_  P_HHF1_-ERG20-F96WN127W-T_SSA1_  P_RPL18b_-t67-*Mc*LIS-T_ADH1_  P_HTB2_-*Ob*GES-T_PGK1_ | This study |
| JBEI-14983 | JBEI-13510 | *ade2*::P_ADE2_-ADE2-T_ADE2_  P_ALD6_-tHMG1-T_ENO1_  P_HHF1_-ERG20-F96WN127W-T_SSA1_  P_RPL18b_-t67-*Mc*LIS-T_ADH1_  P_CCW12_-*Ob*GES-T_PGK1_ | This study |
| JBEI-14988 | JBEI-13510 | *ade2*::P_ADE2_-ADE2-T_ADE2_  P_TEF2_-tHMG1-T_ENO1_  P_HHF1_-ERG20-F96WN127W-T_SSA1_  P_RNR2_-t67-*Mc*LIS-T_ADH1_  P_RNR1_-*Ob*GES-T_PGK1_ | This study |
| JBEI-14989 | JBEI-13510 | *ade2*::P_ADE2_-ADE2-T_ADE2_  P_TEF2_-tHMG1-T_ENO1_  P_HHF1_-ERG20-F96WN127W-T_SSA1_  P_RNR2_-t67-*Mc*LIS-T_ADH1_  P_HTB2_-*Ob*GES-T_PGK1_ | This study |
| JBEI-14990 | JBEI-13510 | *ade2*::P_ADE2_-ADE2-T_ADE2_  P_TEF2_-tHMG1-T_ENO1_  P_PAB1_-ERG20-F96WN127W-T_SSA1_  P_RPL18b_-t67-*Mc*LIS-T_ADH1_  P_HTB2_-*Ob*GES-T_PGK1_ | This study |
| JBEI-15262 | JBEI-13510 | *ade2*::P_ADE2_-ADE2-T_ADE2_  P_TEF2_-tHMG1-T_ENO1_  P_PGK1_-ERG20-F96WN127W-T_SSA1_  P_RPL18b_-t67-*Mc*LIS-T_ADH1_  P_RNR1_-*Ob*GES-T_PGK1_ | This study |
| JBEI-15263 | JBEI-13510 | *ade2*::P_ADE2_-ADE2-T_ADE2_  P_ALD6_-tHMG1-T_ENO1_  P_PGK1_-ERG20-F96WN127W-T_SSA1_  P_HHF2_-t67-*Mc*LIS-T_ADH1_  P_CCW12_-*Ob*GES-T_PGK1_ | This study |

# **Supplementary Table 13** Strain names and genotypes of second iteration strains

| Strain | Parent strain | Description | Reference |
| --- | --- | --- | --- |
| JBEI-16095 | JBEI-13510 | *ade2*::P_ADE2_-ADE2-T_ADE2_  P_CCW12_-tHMG1-T_ENO1_  P_PGK1_-ERG20-F96WN127W-T_SSA1_  P_TEF2_-t67-*Mc*LIS-T_ADH1_  P_HHF2_-*Ob*GES-T_PGK1_ | This study |
| JBEI-16096 | JBEI-13510 | *ade2*::P_ADE2_-ADE2-T_ADE2_  P_TDH3_-tHMG1-T_ENO1_  P_HHF1_-ERG20-F96WN127W-T_SSA1_  P_RPL18b_-t67-*Mc*LIS-T_ADH1_  P_CCW12_-*Ob*GES-T_PGK1_ | This study |
| JBEI-16097 | JBEI-13510 | *ade2*::P_ADE2_-ADE2-T_ADE2_  P_TDH3_-tHMG1-T_ENO1_  P_HHF2_-ERG20-F96WN127W-T_SSA1_  P_HTB2_-t67-*Mc*LIS-T_ADH1_  P_TEF2_-*Ob*GES-T_PGK1_ | This study |
| JBEI-16098 | JBEI-13510 | *ade2*::P_ADE2_-ADE2-T_ADE2_  P_TEF2_-tHMG1-T_ENO1_  P_TEF1_-ERG20-F96WN127W-T_SSA1_  P_HTB2_-t67-*Mc*LIS-T_ADH1_  P_TDH3_-*Ob*GES-T_PGK1_ | This study |
| JBEI-16099 | JBEI-13510 | *ade2*::P_ADE2_-ADE2-T_ADE2_  P_PGK1_-tHMG1-T_ENO1_  P_HHF1_-ERG20-F96WN127W-T_SSA1_  P_TEF1_-t67-*Mc*LIS-T_ADH1_  P_TEF2_-*Ob*GES-T_PGK1_ | This study |
| JBEI-16598 | JBEI-13510 | *ade2*::P_ADE2_-ADE2-T_ADE2_  P_TDH3_-tHMG1-T_ENO1_  P_CCW12_-ERG20-F96WN127W-T_SSA1_  P_TEF1_-t67-*Mc*LIS-T_ADH1_  P_TEF2_-*Ob*GES-T_PGK1_ | This study |
| JBEI-16599 | JBEI-13510 | *ade2*::P_ADE2_-ADE2-T_ADE2_  P_HHF2_-tHMG1-T_ENO1_  P_TDH3_-ERG20-F96WN127W-T_SSA1_  P_TEF2_-t67-*Mc*LIS-T_ADH1_  P_CCW12_-*Ob*GES-T_PGK1_ | This study |
| JBEI-16619 | JBEI-13510 | *ade2*::P_ADE2_-ADE2-T_ADE2_  P_TEF2_-tHMG1-T_ENO1_  P_CCW12_-ERG20-F96WN127W-T_SSA1_  P_RPL18b_-t67-*Mc*LIS-T_ADH1_  P_TDH3_-*Ob*GES-T_PGK1_ | This study |
| JBEI-16621 | JBEI-13510 | *ade2*::P_ADE2_-ADE2-T_ADE2_  P_CCW12_-tHMG1-T_ENO1_  P_PGK1_-ERG20-F96WN127W-T_SSA1_  P_TEF1_-t67-*Mc*LIS-T_ADH1_  P_TDH3_-*Ob*GES-T_PGK1_ | This study |
| JBEI-16645 | JBEI-13510 | *ade2*::P_ADE2_-ADE2-T_ADE2_  P_CCW12_-tHMG1-T_ENO1_  P_HHF1_-ERG20-F96WN127W-T_SSA1_  P_TEF1_-t67-*Mc*LIS-T_ADH1_  P_TDH3_-*Ob*GES-T_PGK1_ | This study |
| JBEI-16652 | JBEI-13510 | *ade2*::P_ADE2_-ADE2-T_ADE2_  P_CCW12_-tHMG1-T_ENO1_  P_TEF1_-ERG20-F96WN127W-T_SSA1_  P_HTB2_-t67-*Mc*LIS-T_ADH1_  P_HHF2_-*Ob*GES-T_PGK1_ | This study |
| JBEI-16654 | JBEI-13510 | *ade2*::P_ADE2_-ADE2-T_ADE2_  P_CCW12_-tHMG1-T_ENO1_  P_HHF1_-ERG20-F96WN127W-T_SSA1_  P_RPL18b_-t67-*Mc*LIS-T_ADH1_  P_HHF2_-*Ob*GES-T_PGK1_ | This study |
| JBEI-16661 | JBEI-13510 | *ade2*::P_ADE2_-ADE2-T_ADE2_  P_TEF2_-tHMG1-T_ENO1_  P_HHF2_-ERG20-F96WN127W-T_SSA1_  P_RPL18b_-t67-*Mc*LIS-T_ADH1_  P_TDH3_-*Ob*GES-T_PGK1_ | This study |
| JBEI-16663 | JBEI-13510 | *ade2*::P_ADE2_-ADE2-T_ADE2_  P_TEF2_-tHMG1-T_ENO1_  P_HHF1_-ERG20-F96WN127W-T_SSA1_  P_HHF2_-t67-*Mc*LIS-T_ADH1_  P_CCW12_-*Ob*GES-T_PGK1_ | This study |
| JBEI-16664 | JBEI-13510 | *ade2*::P_ADE2_-ADE2-T_ADE2_  P_TDH3_-tHMG1-T_ENO1_  P_PAB1_-ERG20-F96WN127W-T_SSA1_  P_HHF2_-t67-*Mc*LIS-T_ADH1_  P_CCW12_-*Ob*GES-T_PGK1_ | This study |
| JBEI-16666 | JBEI-13510 | *ade2*::P_ADE2_-ADE2-T_ADE2_  P_HHF2_-tHMG1-T_ENO1_  P_TEF1_-ERG20-F96WN127W-T_SSA1_  P_HTB2_-t67-*Mc*LIS-T_ADH1_  P_TDH3_-*Ob*GES-T_PGK1_ | This study |
| JBEI-16667 | JBEI-13510 | *ade2*::P_ADE2_-ADE2-T_ADE2_  P_PGK1_-tHMG1-T_ENO1_  P_CCW12_-ERG20-F96WN127W-T_SSA1_  P_TEF2_-t67-*Mc*LIS-T_ADH1_  P_HHF2_-*Ob*GES-T_PGK1_ | This study |
| JBEI-16668 | JBEI-13510 | *ade2*::P_ADE2_-ADE2-T_ADE2_  P_TDH3_-tHMG1-T_ENO1_  P_HHF1_-ERG20-F96WN127W-T_SSA1_  P_TEF2_-t67-*Mc*LIS-T_ADH1_  P_HHF2_-*Ob*GES-T_PGK1_ | This study |
| JBEI-16669 | JBEI-13510 | *ade2*::P_ADE2_-ADE2-T_ADE2_  P_PGK1_-tHMG1-T_ENO1_  P_HHF2_-ERG20-F96WN127W-T_SSA1_  P_HTB2_-t67-*Mc*LIS-T_ADH1_  P_CCW12_-*Ob*GES-T_PGK1_ | This study |
| JBEI-16670 | JBEI-13510 | *ade2*::P_ADE2_-ADE2-T_ADE2_  P_TDH3_-tHMG1-T_ENO1_  P_HHF1_-ERG20-F96WN127W-T_SSA1_  P_TEF1_-t67-*Mc*LIS-T_ADH1_  P_HHF2_-*Ob*GES-T_PGK1_ | This study |
| JBEI-16671 | JBEI-13510 | *ade2*::P_ADE2_-ADE2-T_ADE2_  P_PGK1_-tHMG1-T_ENO1_  P_TEF1_-ERG20-F96WN127W-T_SSA1_  P_CCW12_-t67-*Mc*LIS-T_ADH1_  P_HHF2_-*Ob*GES-T_PGK1_ | This study |
| JBEI-16672 | JBEI-13510 | *ade2*::P_ADE2_-ADE2-T_ADE2_  P_TDH3_-tHMG1-T_ENO1_  P_PGK1_-ERG20-F96WN127W-T_SSA1_  P_HHF2_-t67-*Mc*LIS-T_ADH1_  P_RNR1_-*Ob*GES-T_PGK1_ | This study |
| JBEI-16673 | JBEI-13510 | *ade2*::P_ADE2_-ADE2-T_ADE2_  P_PGK1_-tHMG1-T_ENO1_  P_TDH3_-ERG20-F96WN127W-T_SSA1_  P_RPL18b_-t67-*Mc*LIS-T_ADH1_  P_HHF2_-*Ob*GES-T_PGK1_ | This study |
| JBEI-16674 | JBEI-13510 | *ade2*::P_ADE2_-ADE2-T_ADE2_  P_TEF2_-tHMG1-T_ENO1_  P_CCW12_-ERG20-F96WN127W-T_SSA1_  P_TEF1_-t67-*Mc*LIS-T_ADH1_  P_TDH3_-*Ob*GES-T_PGK1_ | This study |
| JBEI-16676 | JBEI-13510 | *ade2*::P_ADE2_-ADE2-T_ADE2_  P_HHF2_-tHMG1-T_ENO1_  P_CCW12_-ERG20-F96WN127W-T_SSA1_  P_RPL18b_-t67-*Mc*LIS-T_ADH1_  P_TEF2_-*Ob*GES-T_PGK1_ | This study |
| JBEI-16677 | JBEI-13510 | *ade2*::P_ADE2_-ADE2-T_ADE2_  P_TEF2_-tHMG1-T_ENO1_  P_HHF2_-ERG20-F96WN127W-T_SSA1_  P_TEF1_-t67-*Mc*LIS-T_ADH1_  P_CCW12_-*Ob*GES-T_PGK1_ | This study |
| JBEI-16679 | JBEI-13510 | *ade2*::P_ADE2_-ADE2-T_ADE2_  P_HHF2_-tHMG1-T_ENO1_  P_CCW12_-ERG20-F96WN127W-T_SSA1_  P_TEF2_-t67-*Mc*LIS-T_ADH1_  P_TDH3_-*Ob*GES-T_PGK1_ | This study |
| JBEI-16680 | JBEI-13510 | *ade2*::P_ADE2_-ADE2-T_ADE2_  P_TEF2_-tHMG1-T_ENO1_  P_TEF1_-ERG20-F96WN127W-T_SSA1_  P_HTB2_-t67-*Mc*LIS-T_ADH1_  P_HHF2_-*Ob*GES-T_PGK1_ | This study |
| JBEI-16681 | JBEI-13510 | *ade2*::P_ADE2_-ADE2-T_ADE2_  P_HHF2_-tHMG1-T_ENO1_  P_TDH3_-ERG20-F96WN127W-T_SSA1_  P_RPL18b_-t67-*Mc*LIS-T_ADH1_  P_CCW12_-*Ob*GES-T_PGK1_ | This study |
| JBEI-16682 | JBEI-13510 | *ade2*::P_ADE2_-ADE2-T_ADE2_  P_TEF2_-tHMG1-T_ENO1_  P_PGK1_-ERG20-F96WN127W-T_SSA1_  P_TEF1_-t67-*Mc*LIS-T_ADH1_  P_HHF2_-*Ob*GES-T_PGK1_ | This study |

#

# **Supplementary Table 14** Monoterpene concentrations after fermentation as measured by GCMS

| Strain | Linalool (mg/L) | Standard deviation (Linalool) | Geraniol (mg/L) | Standard deviation (Geraniol) |
| --- | --- | --- | --- | --- |
| JBEI-14953 | 3.843 | 0.556 | 0.503 | 0.086 |
| JBEI-14954 | 0.162 | 0.012 | 0.543 | 0.062 |
| JBEI-14955 | 0.115 | 0.012 | 0.248 | 0.061 |
| JBEI-14968 | 0.067 | 0.032 | 0.380 | 0.040 |
| JBEI-14969 | 0.294 | 0.132 | 0.101 | 0.038 |
| JBEI-14970 | 0.338 | 0.028 | 0.405 | 0.031 |
| JBEI-14971 | 0.089 | 0.020 | 0.083 | 0.021 |
| JBEI-14972 | 0.164 | 0.022 | 0.224 | 0.027 |
| JBEI-14973 | 0.021 | 0.005 | 0.048 | 0.005 |
| JBEI-14974 | 0.054 | 0.004 | 0.083 | 0.011 |
| JBEI-14975 | 0.054 | 0.019 | 0.073 | 0.030 |
| JBEI-14976 | 0.065 | 0.002 | 0.094 | 0.002 |
| JBEI-14983 | 0.004 | 0.001 | 0.008 | 0.008 |
| JBEI-14988 | 0.001 | 0.000 | 0.011 | 0.019 |
| JBEI-14989 | 0.001 | 0.000 | 0.052 | 0.025 |
| JBEI-14990 | 0.076 | 0.027 | 0.003 | 0.000 |
| JBEI-15262 | 0.167 | 0.059 | 0.115 | 0.043 |
| JBEI-15263 | 0.354 | 0.030 | 0.003 | 0.000 |
| JBEI-16095 | 0.274 | 0.013 | 0.090 | 0.046 |
| JBEI-16096 | 0.040 | 0.001 | 0.030 | 0.007 |
| JBEI-16097 | 0.029 | 0.020 | 0.069 | 0.059 |
| JBEI-16098 | 0.261 | 0.015 | 0.433 | 0.054 |
| JBEI-16099 | 0.463 | 0.269 | 0.149 | 0.215 |
| JBEI-16598 | 0.478 | 0.019 | 0.369 | 0.022 |
| JBEI-16599 | 0.469 | 0.024 | 0.461 | 0.010 |
| JBEI-16619 | 1.011 | 0.051 | 1.453 | 0.060 |
| JBEI-16621 | 0.626 | 0.041 | 0.493 | 0.035 |
| JBEI-16645 | 0.267 | 0.032 | 0.036 | 0.006 |
| JBEI-16652 | 0.104 | 0.068 | 0.480 | 0.018 |
| JBEI-16654 | 0.048 | 0.053 | 0.005 | 0.008 |
| JBEI-16661 | 0.022 | 0.002 | 0.001 | 0.000 |
| JBEI-16663 | 0.633 | 0.024 | 0.528 | 0.062 |
| JBEI-16664 | 0.162 | 0.150 | 0.002 | 0.000 |
| JBEI-16666 | 0.248 | 0.033 | 0.571 | 0.089 |
| JBEI-16667 | 3.584 | 1.036 | 1.428 | 0.379 |
| JBEI-16668 | 0.190 | 0.011 | 0.001 | 0.000 |
| JBEI-16669 | 0.117 | 0.074 | 0.444 | 0.108 |
| JBEI-16670 | 0.112 | 0.003 | 0.001 | 0.000 |
| JBEI-16671 | 1.418 | 0.034 | 0.043 | 0.001 |
| JBEI-16672 | 0.595 | 0.083 | 0.001 | 0.000 |
| JBEI-16673 | 0.246 | 0.262 | 0.214 | 0.123 |
| JBEI-16674 | 0.774 | 0.036 | 0.042 | 0.003 |
| JBEI-16676 | 0.295 | 0.030 | 0.704 | 0.018 |
| JBEI-16677 | 0.614 | 0.015 | 0.388 | 0.029 |
| JBEI-16679 | 0.558 | 0.018 | 0.209 | 0.110 |
| JBEI-16680 | 0.506 | 0.043 | 0.856 | 0.068 |
| JBEI-16681 | 0.193 | 0.019 | 0.573 | 0.075 |
| JBEI-16682 | 0.882 | 0.073 | 0.173 | 0.089 |
| Iteration 1 Centroid | 0.326 | 0.053 | 0.165 | 0.028 |
| Iteration 2  Centroid | 0.507 | 0.085 | 0.341 | 0.056 |

# **Supplementary Table 15** Fermentable sugars remaining and ethanol produced after fermentation as measured by HPLC

| Strain | Maltotriose (g/L) | Maltose (g/L) | Glucose (g/L) | Ethanol (g/L) |
| --- | --- | --- | --- | --- |
| JBEI-14953 | 6.618 | 19.057 | 0.0047 | 14.348 |
| JBEI-14954 | 2.853 | 3.463 | 0.1124 | 26.659 |
| JBEI-14955 | 0.232 | 0.795 | 0.0047 | 23.009 |
| JBEI-14968 | 2.335 | 2.841 | 0.0360 | 15.534 |
| JBEI-14969 | 0.905 | 2.081 | 0.0138 | 23.174 |
| JBEI-14970 | 7.339 | 21.372 | 0.0149 | 10.633 |
| JBEI-14971 | 0.009 | 0.840 | 0.0047 | 20.583 |
| JBEI-14972 | 0.595 | 1.026 | 0.0047 | 24.260 |
| JBEI-14973 | 6.898 | 19.588 | 0.1330 | 11.125 |
| JBEI-14974 | 2.869 | 2.791 | 0.0047 | 24.060 |
| JBEI-14975 | 2.591 | 2.890 | 0.0047 | 22.274 |
| JBEI-14976 | 1.202 | 2.925 | 0.0047 | 25.090 |
| JBEI-14983 | 3.684 | 4.091 | 0.0123 | 22.964 |
| JBEI-14988 | 0.815 | 1.177 | 0.1102 | 25.436 |
| JBEI-14989 | 0.009 | 0.169 | 0.0148 | 26.118 |
| JBEI-14990 | 1.884 | 2.118 | 0.0047 | 22.028 |
| JBEI-15262 | 4.227 | 5.019 | 0.0125 | 21.910 |
| JBEI-15263 | 0.009 | 1.072 | 0.0177 | 26.931 |
| JBEI-16095 | 3.354 | 1.965 | 0.0003 | 33.710 |
| JBEI-16096 | 3.278 | 1.790 | 0.0003 | 32.416 |
| JBEI-16097 | 3.797 | 2.574 | 0.0003 | 30.510 |
| JBEI-16098 | 3.545 | 2.319 | 0.0003 | 32.954 |
| JBEI-16099 | 5.482 | 7.381 | 0.0003 | 30.388 |
| JBEI-16598 | 3.170 | 1.671 | 0.0003 | 35.054 |
| JBEI-16599 | 3.591 | 2.551 | 0.0003 | 31.833 |
| JBEI-16619 | 3.439 | 2.129 | 0.0003 | 33.768 |
| JBEI-16621 | 2.762 | 0.820 | 0.0003 | 34.237 |
| JBEI-16645 | 3.123 | 1.648 | 0.0003 | 31.893 |
| JBEI-16652 | 3.656 | 2.353 | 0.0003 | 33.348 |
| JBEI-16654 | 8.422 | 23.631 | 0.0003 | 18.197 |
| JBEI-16661 | 3.157 | 1.600 | 0.0003 | 33.361 |
| JBEI-16663 | 3.172 | 1.779 | 0.0003 | 31.887 |
| JBEI-16664 | 3.311 | 2.148 | 0.0003 | 30.959 |
| JBEI-16666 | 3.083 | 1.693 | 0.0003 | 33.701 |
| JBEI-16667 | 4.310 | 1.498 | 0.0003 | 34.826 |
| JBEI-16668 | 4.965 | 6.231 | 0.0003 | 29.774 |
| JBEI-16669 | 3.291 | 1.855 | 0.0003 | 35.041 |
| JBEI-16670 | 2.873 | 1.362 | 0.0003 | 34.791 |
| JBEI-16671 | 3.598 | 2.721 | 0.0003 | 34.560 |
| JBEI-16672 | 3.493 | 2.585 | 0.0003 | 31.437 |
| JBEI-16673 | 4.197 | 3.768 | 0.0003 | 33.124 |
| JBEI-16674 | 3.683 | 2.429 | 0.0003 | 33.242 |
| JBEI-16676 | 3.188 | 1.807 | 0.0003 | 34.068 |
| JBEI-16677 | 4.546 | 3.870 | 0.0003 | 32.554 |
| JBEI-16679 | 2.997 | 1.646 | 0.0003 | 33.865 |
| JBEI-16680 | 4.062 | 3.097 | 0.0003 | 32.429 |
| JBEI-16681 | 3.700 | 3.024 | 0.0003 | 31.154 |
| JBEI-16682 | 3.536 | 2.287 | 0.0003 | 34.449 |

#

# **Supplementary Table 16** Protein abundance (total protein area)

| Strain | tHMGR | FPPS* | Linalool synthase | Geraniol synthase |
| --- | --- | --- | --- | --- |
| JBEI-14953 | 46294.300 | 61749.710 | 7402.689 | 694.593 |
| JBEI-14954 | 16710.980 | 30271.121 | 1362.031 | 2080.742 |
| JBEI-14955 | 13453.780 | 26290.896 | 3551.335 | 3696.098 |
| JBEI-14968 | 5795.770 | 9286.386 | 336.350 | 1745.693 |
| JBEI-14969 | 8778.641 | 22922.219 | 2464.120 | 402.745 |
| JBEI-14970 | 14127.300 | 32855.923 | 2958.891 | 3091.251 |
| JBEI-14971 | 6758.793 | 9092.046 | 1147.474 | 417.553 |
| JBEI-14972 | 9571.769 | 10945.530 | 2142.471 | 1906.866 |
| JBEI-14973 | 19389.671 | 10879.197 | 568.186 | 230.264 |
| JBEI-14974 | 15704.779 | 8995.311 | 1574.220 | 377.276 |
| JBEI-14975 | 9319.016 | 14119.950 | 1269.566 | 438.467 |
| JBEI-14976 | 11020.566 | 14877.566 | 1391.894 | 512.985 |
| JBEI-14983 | 2983.832 | 14838.114 | 1185.394 | 1718.830 |
| JBEI-14988 | 5754.331 | 9356.943 | 365.620 | 237.955 |
| JBEI-14989 | 6356.994 | 11020.338 | 208.094 | 465.921 |
| JBEI-14990 | 8227.666 | 8338.383 | 777.102 | 405.419 |
| JBEI-15262 | 9743.995 | 18081.904 | 1503.451 | 295.152 |
| JBEI-15263 | 2900.501 | 23202.188 | 3124.680 | 2512.464 |
| JBEI-16095 | 20474.100 | 17711.137 | 8545.443 | 622.860 |
| JBEI-16096 | 19124.540 | 19994.175 | 2997.740 | 9172.155 |
| JBEI-16097 | 18091.790 | 18404.273 | 2563.367 | 3787.273 |
| JBEI-16098 | 17514.190 | 31567.677 | 1916.223 | 2496.267 |
| JBEI-16099 | 17369.357 | 18501.317 | 9925.830 | 3490.040 |
| JBEI-16598 | 19610.067 | 37980.823 | 7472.970 | 3524.570 |
| JBEI-16599 | 13396.880 | 57354.287 | 12314.740 | 5166.850 |
| JBEI-16619 | 16206.527 | 45602.563 | 2782.870 | 3451.343 |
| JBEI-16621 | 24364.443 | 29458.623 | 11062.143 | 2999.473 |
| JBEI-16645 | 19822.733 | 17402.283 | 10495.493 | 2806.700 |
| JBEI-16652 | 26006.783 | 28662.457 | 1296.853 | 1501.493 |
| JBEI-16654 | 20618.583 | 18952.213 | 3097.653 | 1883.330 |
| JBEI-16661 | 17359.100 | 27385.927 | 3907.510 | 4477.920 |
| JBEI-16663 | 16625.343 | 14240.707 | 6036.790 | 5295.740 |
| JBEI-16664 | 52963.143 | 23671.387 | 13908.027 | 11244.240 |
| JBEI-16666 | 10348.877 | 30232.160 | 3044.643 | 4665.903 |
| JBEI-16667 | 14089.147 | 31531.993 | 6465.207 | 802.990 |
| JBEI-16668 | 18744.913 | 15292.113 | 8482.073 | 1011.540 |
| JBEI-16669 | 13195.290 | 18793.995 | 1910.315 | 7660.925 |
| JBEI-16670 | 20967.203 | 19471.620 | 10985.173 | 2185.243 |
| JBEI-16671 | 18254.780 | 25980.673 | 7154.360 | 2727.577 |
| JBEI-16672 | 51190.310 | 45493.357 | 12366.913 | 965.126 |
| JBEI-16673 | 11475.077 | 17722.067 | 1250.893 | 550.480 |
| JBEI-16674 | 14500.283 | 41299.713 | 8351.270 | 1704.693 |
| JBEI-16676 | 11254.683 | 61582.137 | 3897.487 | 5965.893 |
| JBEI-16677 | 10083.613 | 12135.973 | 6239.280 | 2339.720 |
| JBEI-16679 | 13516.547 | 60292.777 | 13508.283 | 3775.027 |
| JBEI-16680 | 23179.263 | 54941.557 | 2750.157 | 3207.057 |
| JBEI-16681 | 13192.357 | 61378.780 | 3676.633 | 7408.330 |
| JBEI-16682 | 21734.797 | 38277.207 | 9907.590 | 1267.464 |

#

# **Supplementary Table 17** Alcolyzer measurements of industrial fermentations (first set)

| Sample | Apparent extract (%m/m) | Real extract (%m/m) | Alcohol (%V/V) | Density (g/cm^3^) | Specific gravity | RDF (%) |
| --- | --- | --- | --- | --- | --- | --- |
| WLP001 | 2.13 | 3.96 | 5.03 | 1.0065 | 1.00831 | 67.3 |
| JBEI-14971 | 2.7 | 4.38 | 4.64 | 1.0087 | 1.01051 | 63.14 |
| JBEI-16669 | 2.92 | 4.59 | 4.58 | 1.0096 | 1.01142 | 61.72 |
| JBEI-16652 | 4.22 | 5.61 | 3.84 | 1.0147 | 1.01653 | 52.39 |

# **Supplementary Table 18** Alcolyzer measurements of industrial fermentations (second set)

| Sample | Apparent extract (%m/m) | Real extract (%m/m) | Alcohol (%V/V) | Density (g/cm^3^) | Specific gravity | RDF (%) |
| --- | --- | --- | --- | --- | --- | --- |
| WLP001 | 2.19 | 3.94 | 4.81 | 1.0067 | 1.00851 | 66.44 |
| JBEI-16652 | 3.71 | 5.13 | 3.89 | 1.0127 | 1.01452 | 54.96 |
| WLP001 WA dry hop | 2.26 | 3.89 | 4.45 | 1.007 | 1.00881 | 64.97 |
| WLP001 ID dry hop | 2.31 | 4.01 | 4.67 | 1.0072 | 1.00901 | 65.32 |

**Supplementary Note 1**

**Developing a methodology for stable and markerless integration in brewer’s yeast.**

In preliminary experiments the rate of stable pathway integration was considerably lower than that previously reported for gene deletions in other industrial yeast strains[^17,18^](https://paperpile.com/c/3CUptU/Zl1XX+BBbqb). This could be explained by methodological differences or by the extended repair template size. To assist with the challenge of identifying stable pathway transformants, the integration strategy leverages a colorimetric assay commonly used in yeast genetic screens[^19,20^](https://paperpile.com/c/3CUptU/GpGJl+YVOVM). That is, a yeast strain lacking the ADE2 gene accumulates a red intermediate derived from the adenine biosynthesis pathway. For the strain construction strategy, an *ade2* deletion strain was generated from the parent strain and a copy of the ADE2 gene was included in the repair template as part of the 5’ homology arm (Fig. 2a). Therefore, transformants with on-target pathway integration appeared white on a transformation plate, while unsuccessful transformants remained red (Fig. 2b). In order to confirm that all genetic copies of the ADE2 locus were successfully replaced with the biosynthetic pathway gene construct, integration events were confirmed by diagnostic PCR. Results revealed that a fraction of the white transformants were heterozygous for the biosynthetic pathway genes at the ADE2 locus. Interestingly, the heterozygous colonies exhibited a variegated phenotype upon further propagation (Fig. 2c). This serendipitous finding not only illustrates the instability of heterozygote genetic modifications in industrial strains, but also highlights a feature of the colorimetric assay that can be used to distinguish unstable heterozygous transformants from stable homozygous transformants. Notably, when red colonies from the variegated patch were restreaked, resulting colonies remained red, but when white colonies from the variegated patch were restreaked, resulting colonies exhibited the variegated phenotype (data not shown). Thus the heterozygous strains exhibit a high rate of loss of heterozygosity (LOH), and that LOH mutants were biased towards loss of heterologous pathway genes, rather than gain of heterologous pathway genes.

**Supplementary Note 2**

**Fermentable sugar consumption.**

Several engineered strains exhibited incomplete consumption of fermentable sugars (Supplementary Fig. 6). There are several potential explanations for this observation based on previous studies.

Firstly, heterologous protein expression can introduce a metabolic burden by overloading DNA replication machinery and/or protein expression machinery[^21,22^](https://paperpile.com/c/3CUptU/WnQZf+FvBW7). This is not a likely explanation in the present study. The integrated DNA amounts to a very small percentage of the total genome size (~0.1%) and the heterologous protein expression is small relative to the expression of the total proteome (~0.03%-0.09% of the total proteome). In addition, no significant correlation was observed between relative heterologous protein expression and remaining fermentable sugar (correlation of 0.27, p-value of 0.02). Finally, we measured the rate of sugar consumption over a 5-day fermentation for the strain predicted to drive highest expression of heterologous proteins and observed indistinguishable growth properties compared to the parent strain (Supplementary Fig. 7).

Secondly, end-product accumulation can lead to cellular toxicity. Notably, previous studies have reported toxicity of monoterpenes at relatively low concentrations in *S. cerevisiae* due to cell wall stress[^23,24^](https://paperpile.com/c/3CUptU/JWZXd+MUFm2). We therefore measured growth inhibition at various concentrations of linalool and geraniol (Supplementary Fig. 12). The concentrations of linalool and geraniol produced in the engineered strains were well below the minimum concentration of growth inhibition, suggesting that accumulation of these molecules at concentrations characteristic of finished beer does not adversely affect fermentation. We did not observe a significant correlation between remaining fermentable sugars and total monoterpene production (correlation of 0.28, *p*-value of 0.26), suggesting sugar consumption defects stemmed from pathway imbalance rather than metabolic burden.

Thirdly, metabolic pathway imbalance can lead to accumulation of toxic intermediates. These defects might be explained by imbalances in pathway enzyme expression that lead to increased accumulation of metabolite intermediates, as certain intermediates have been implicated as toxic at high cellular levels in various microorganisms[^25^](https://paperpile.com/c/3CUptU/8zLkL), including yeast[^26^](https://paperpile.com/c/3CUptU/SvdVO).

**Supplementary Note 3**

**Details of model development and performance evaluation.**

To establish a framework with which we could evaluate prospective genetic designs, we developed three mathematical models. Structured to contain a varying degree of prior biological knowledge, these models were used to predict the relationship between protein abundance and monoterpene production. Unstructured models, such as those used in deep-learning algorithms, are powerful in their capacity to capture complex biological relationships in large datasets. However, biologically-informed models may better capture quantitative relationships for qualitatively predictable biological phenomena in smaller datasets. Interestingly, the most biologically-informed model—based on Michaelis-Menten enzyme kinetics—best predicted actual monoterpene concentrations for the amount and type of data available.

The three models used in this work are a Gaussian regressor, a linear model, and a kinetic model. The Gaussian regressor model contains no biological intuition and is purely a sophisticated interpolation scheme. The linear model incorporates two pieces of biological intuition gleaned from the data and uses them to create a heuristic model that extrapolates well. The kinetic model is a detailed Michaelis-Menten-based model that uses in vitro kinetic coefficients from the literature where available. This model incorporates the most biological intuition and is derived almost strictly from the literature. Each model is described in more detail as follows:

**The Gaussian regressor model** is implemented in Scikit Learn and is the most 'off the shelf' of the models we used. Standard best practices for machine learning were used to validate and build the model. This model uses no biological intuition and is conceptually similar to an interpolation scheme. As this experiment was in vivo, the data may be influenced by more variables than what can intuitively be captured with biological intuition. Our thinking was that an unconstrained model may perform better than one constrained by a limited biological intuition. This seems to be the case with large data sets. With small data sets, however, models that include more biological intuition perform better. Like all of the models analyzed, it was validated using leave one out cross-validation on the first cycle of data.

**The linear model** incorporates the two design principles gleaned from the original data set and combines them together into a composite model that does well at predicting linalool and geraniol from the proteomics data. The first observation was that FPPS and tHMGR are both positively correlated with increasing total terpene titer. Second, the titer-normalized ratio of linalool to geraniol was predicted by the ratio of LIS to GES. As a result, we trained models to learn to predict total terpene titer from tHMGR and FPPS and the ratio of linalool to geraniol from the ratio of LIS to GES. Using all proteins, we can predict both total terpene titer and individual linalool and geraniol titers. Multiplying total titer by the percentage of linalool will yield total linalool titer. Similarly, multiplying total titer by the percentage of geraniol will yield total geraniol titer. Mathematically the predictions of these models can be found by:

 $Total terpene production =\alpha_{1}[tHMGR]+\alpha_{2}[FPPS]+\alpha_{3}$ (2)
 $Percent geraniol production =\alpha_{4}[LIS]+\alpha_{5}[GES]+\alpha_{6}$ (3)
 $Percent linalool production =\alpha_{7}[LIS]+\alpha_{8}[GES]+\alpha_{9}$ (4)
 $[Linalool]=Total terpene production*Percent linalool production$ (5)
 $[Geraniol]=Total terpene production*Percent geraniol production$ (6)

The coefficients $\alpha_{i}$ above were calculated using linear machine learning models implemented in Scikit Learn. The composite model was validated using leave one out cross-validation. The moments of the error residuals of the model are presented in Fig. 2.

**The kinetic model** developed here uses Michaelis-Menten kinetics together with information from the literature about the mevalonate pathway. This pathway has been heavily studied and so there are kinetic coefficients that were used as a starting point to parameterize the model. Our goal was to strike a balance between the number of free parameters in the model and the amount of detail. Increasing the number of free parameters (which come from additional sources of biological intuition) could lead to over-parameterizing the model. Ultimately, too many parameters with a small amount of data will make the model generalize poorly. Additionally, to avoid over-fitting, the model was cross-validated using a leave one out methodology. This model is subject to a few assumptions.

First, we assume that the intermediate metabolites do not cause feedback inhibition. This is reasonable because only HMGR appears to be feedback inhibited and this was addressed in strain engineering. A truncated HMGR that does not suffer from inhibition, tHMGR, was engineered into the host. Additionally, the goal is not to maximize production, but rather to hit a particular target to mimic the taste of hops in beer. As a result, feedback inhibition is much less of a concern than it would be if we were trying to maximize titers of linalool and geraniol. Second, we assume that IPP is an abundant enough reactant to not be included in the model. Third, tHMGR is assumed to be the rate-limiting step in a sequence of steps leading to GPP synthesis. To simplify the model, this entire linear sequence of steps was modeled by a single Michaelis-Menten kinetic equation that accurately describes the flux under tested conditions.

Both a native and a heterologous FPPS were present in the engineered host. This enzyme catalyzes two steps converting DMAPP and IPP into GPP, then GPP and IPP into FPP. The native enzyme has a quick conversion of GPP to FPP, whereas the engineered enzyme only slowly catalyzes that step. Since GPP is the precursor to both linalool and geraniol, this element is important to capture in the model. Together these assumptions and observations were used to craft the following system, which describes the pathway dynamics.

$\frac{d[DMAPP]}{dt}= \frac{K_{cat6}\left[ tHMGR \right][HMG-CoA]}{K_{m6}+[HMG-CoA]}- \frac{K_{cat0}\left[ {FPPS}_{1} \right]\left[ DMAPP \right]}{K_{m0}+\left[ DMAPP \right]}- \frac{K_{cat1}\left[ {FPPS}_{2} \right][DMAPP]}{K_{m1}+[DMAPP]}$ (7)

$\frac{d\left[ GPP \right]}{dt}= \frac{K_{cat0}\left[ {FPPS}_{1} \right]\left[ DMAPP \right]}{K_{m0}+\left[ DMAPP \right]}+ \frac{K_{cat1}\left[ {FPPS}_{2} \right]\left[ DMAPP \right]}{K_{m1}+\left[ DMAPP \right]}- \frac{K_{cat2}\left[ {FPPS}_{1} \right]\left[ GPP \right]}{K_{m2}+\left[ GPP \right]}$

$- \frac{K_{cat3}\left[ {FPPS}_{2} \right]\left[ GPP \right]}{K_{m3}+\left[ GPP \right]}- \frac{K_{cat4}\left[ LIS \right]\left[ GPP \right]}{K_{m4}+\left[ GPP \right]}- \frac{K_{cat5}\left[ GES \right][GPP]}{K_{m5}+[GPP]}$ (8)

$\frac{d[Linalool]}{dt}= \frac{K_{cat4}\left[ LIS \right][GPP]}{K_{m4}+[GPP]}$ (9)

$\frac{d[Geraniol]}{dt}= \frac{K_{cat5}\left[ GES \right][GPP]}{K_{m5}+[GPP\}}$ (10)

The following components are modeled in the system: tHMGR, FPPS1, FPPS2, LIS, GES, IPP, DMAPP, GPP, FPP, linalool, geraniol. We assume the system has reached steady state so all nonterminal metabolite concentrations are constant. This means all metabolite derivatives except for linalool and geraniol are zero. Additionally, it is assumed that IPP concentrations are not limiting and it can be excluded from the dynamics of FPPS.

Free parameters are included to convert relative protein counts to absolute proteomics values. Additionally, a parameter $\beta$ determines the relative ratio between the endogenous FPPS and the engineered FPPS. Since FPPS is measured as a combination of the endogenous and engineered versions of the proteins we express [FPPS_1] and [FPPS_2] as functions of FPPS counts and the parameters $\alpha_{0}$ and $\beta$. This is represented in the model by

$\left[ {FPPS}_{2} \right]= \propto_{0}(1-\beta)[FPPS]$ (11)
 $\left[ {FPPS}_{1} \right]= \alpha_{0}\beta[FPPS\}$ (12)

Additionally, kinetic coefficients were scraped from the literature and can be found in Supplementary Table 5. These served as starting points for the model parameters and were allowed to vary over an order of magnitude to improve model fit. We wanted to derive an optimization problem which would allow us to determine the production of linalool and geraniol from [FPPS], [LIS], [GES], [tHMGR]. Given an existing data set, we found the parameters to fit the model. The free parameters include kinetic parameters bounded by the literature, conversion factors for relative to absolute proteomics, and a ratio parameter to fix the ratio of the engineered FPPS to the endogenous FPPS. These parameters are then optimized with respect to the training data set. After parameter optimization, the kinetic model performance is evaluated using leave one out cross-validation.

To evaluate the extent to which the second iteration genetic designs were targeted towards desired terpene biosynthesis levels, we compared simulated monoterpene production for second iteration genetic designs with simulated production for randomly generated strains.

Univariate linear models were created which related genetic toolkit promoters (ranked 1-19) to protein concentrations for each modulated gene in the monoterpene pathway. The full details of the linear model implementation can be found in Supplementary Data File 1. The error residuals of the univariate linear models were used to compute noise for simulated protein levels. This was computed as:

$\Omega\sim N(\mu_{i},\sigma_{i})$ (13)

$\omega\in\Omega$ (14)

${[protein]}_{i}=f_{i}(p_{rank}) +\omega$ (15)

where the function $f_{i}$ corresponds to the model for protein $i$, and $\omega$ is a value generated at random from the error distribution $\Omega$, corresponding to the model for protein $i$. The resulting protein concentrations were then used to compute terpene concentrations and noise was computed based on the error residual distribution from the kinetics-based model as follows:

$\Xi\sim N_{2}(\mu,\Sigma_{i})$ (16)

$\xi\in\Xi$ (17)

$\left[ L,G \right] = g(\left[ tHMGR \right],\left[ FPPS* \right],\left[ LIS \right],\left[ GES \right]) + \xi$ (18)

where $L$ and $G$ are linalool and geraniol concentrations respectively. $g$is the mapping of the kinetics-based model from pathway protein concentrations to terpene concentrations. $\xi$ is a random error residual drawn from the bivariate normal error residual distribution corresponding to the kinetics-based model. Using these equations, a single realization of phenotype predicted from genotype can be created.

To compare the predicted performance of second iterations strains to randomly generated strains, we created a pool of 25000 genotypes that were sampled from the 30 second iteration strains and a pool of 25000 genotypes that were generated by uniformly sampling promoter ranks between values 1 and 19 for each of the four promoter-modulated genes. The improvement of each model over random was evaluated and is presented in Fig. 4 and Supplementary Fig. 10.

# **Supplementary References:**

1. [Kirtley, M. E. & Rudney, H. Some Properties and Mechanism of Action of the β-Hydroxy-β-methylglutaryl Coenzyme A Reductase of Yeast*. *Biochemistry* **6,** 230–238 (1967).](http://paperpile.com/b/3CUptU/nGBhm)

2. [Ignea, C., Pontini, M., Maffei, M. E., Makris, A. M. & Kampranis, S. C. Engineering monoterpene production in yeast using a synthetic dominant negative geranyl diphosphate synthase. *ACS Synth. Biol.* **3,** 298–306 (2014).](http://paperpile.com/b/3CUptU/ZgFwx)

3. [Crowell, A. L., Williams, D. C., Davis, E. M., Wildung, M. R. & Croteau, R. Molecular cloning and characterization of a new linalool synthase. *Arch. Biochem. Biophys.* **405,** 112–121 (2002).](http://paperpile.com/b/3CUptU/cIN53)

4. [Iijima, Y., Gang, D. R., Fridman, E., Lewinsohn, E. & Pichersky, E. Characterization of Geraniol Synthase from the Peltate Glands of Sweet Basil. *Plant Physiol.* **134,** 370–379 (2004).](http://paperpile.com/b/3CUptU/bPm5P)

5. [Pichersky, E., Lewinsohn, E. & Croteau, R. Purification and characterization of S-linalool synthase, an enzyme involved in the production of floral scent in Clarkia breweri. *Arch. Biochem. Biophys.* **316,** 803–807 (1995).](http://paperpile.com/b/3CUptU/Zq9MN)

6. [Chen, X. *et al.* Characterisation of an (S)-linalool synthase from kiwifruit (Actinidia arguta) that catalyses the first committed step in the production of floral lilac compounds. *Funct. Plant Biol.* **37,** 232–243 (2010).](http://paperpile.com/b/3CUptU/EV6Sh)

7. [Hernández, I., Molenaar, D., Beekwilder, J., Bouwmeester, H. & van Hylckama Vlieg, J. E. T. Expression of plant flavor genes in Lactococcus lactis. *Appl. Environ. Microbiol.* **73,** 1544–1552 (2007).](http://paperpile.com/b/3CUptU/NByyB)

8. [Ito, M. & Honda, G. Geraniol synthases from perilla and their taxonomical significance. *Phytochemistry* **68,** 446–453 (2007).](http://paperpile.com/b/3CUptU/3tfSg)

9. [Masumoto, N., Korin, M. & Ito, M. Geraniol and linalool synthases from wild species of perilla. *Phytochemistry* **71,** 1068–1075 (2010).](http://paperpile.com/b/3CUptU/suUyy)

10. [Crowell, A. L., Williams, D. C., Davis, E. M., Wildung, M. R. & Croteau, R. Molecular cloning and characterization of a new linalool synthaseq. *Arch. Biochem. Biophys.* **405,** 112–121 (2002).](http://paperpile.com/b/3CUptU/rXp40)

11. [van Schie, C. C. N., Haring, M. A. & Schuurink, R. C. Tomato linalool synthase is induced in trichomes by jasmonic acid. *Plant Mol. Biol.* **64,** 251–263 (2007).](http://paperpile.com/b/3CUptU/U903r)

12. [Shishido, H. *et al.* Geraniol synthase whose mRNA is induced by host-selective ACT-toxin in the ACT-toxin-insensitive rough lemon (Citrus jambhiri). *J. Plant Physiol.* **169,** 1401–1407 (2012).](http://paperpile.com/b/3CUptU/ojYfJ)

13. [Vezzaro, A. *et al.* Isolation and characterization of terpene synthases potentially involved in flavor development of ripening olive (Olea europaea) fruits. *J. Plant Physiol.* **169,** 908–914 (2012).](http://paperpile.com/b/3CUptU/hj87I)

14. [Dong, L. *et al.* Characterization of two geraniol synthases from Valeriana officinalis and Lippia dulcis: similar activity but difference in subcellular localization. *Metab. Eng.* **20,** 198–211 (2013).](http://paperpile.com/b/3CUptU/kgk9Z)

15. [Martin, D. M. *et al.* Functional Annotation, Genome Organization and Phylogeny of the Grapevine (Vitis vinifera) Terpene Synthase Gene Family Based on Genome Assembly, FLcDNA Cloning, and Enzyme Assays. *BMC Plant Biol.* **10,** 226 (2010).](http://paperpile.com/b/3CUptU/e1WPU)

16. [Iijima, Y., Gang, D. R., Fridman, E., Lewinsohn, E. & Pichersky, E. Characterization of Geraniol Synthase from the Peltate Glands of Sweet Basil. *Plant Physiol.* **134,** 370–379 (2004).](http://paperpile.com/b/3CUptU/klNCw)

17. [Stovicek, V., Borodina, I. & Forster, J. CRISPR–Cas system enables fast and simple genome editing of industrial Saccharomyces cerevisiae strains. *Metabolic Engineering Communications* **2,** 13–22 (2015).](http://paperpile.com/b/3CUptU/Zl1XX)

18. [Zhang, G.-C. *et al.* Construction of a quadruple auxotrophic mutant of an industrial polyploid saccharomyces cerevisiae strain by using RNA-guided Cas9 nuclease. *Appl. Environ. Microbiol.* **80,** 7694–7701 (2014).](http://paperpile.com/b/3CUptU/BBbqb)

19. [Koshland, D., Kent, J. C. & Hartwell, L. H. Genetic analysis of the mitotic transmission of minichromosomes. *Cell* **40,** 393–403 (1985).](http://paperpile.com/b/3CUptU/GpGJl)

20. [Forsburg, S. L. The art and design of genetic screens: yeast. *Nat. Rev. Genet.* **2,** 659–668 (2001).](http://paperpile.com/b/3CUptU/YVOVM)

21. [Bentley, W. E., Mirjalili, N., Andersen, D. C., Davis, R. H. & Kompala, D. S. Plasmid-encoded protein: the principal factor in the ‘metabolic burden’ associated with recombinant bacteria. *Biotechnol. Bioeng.* **35,** 668–681 (1990).](http://paperpile.com/b/3CUptU/WnQZf)

22. [Ajikumar, P. K. *et al.* Isoprenoid pathway optimization for Taxol precursor overproduction in Escherichia coli. *Science* **330,** 70–74 (2010).](http://paperpile.com/b/3CUptU/FvBW7)

23. [Brennan, T. C. R., Turner, C. D., Krömer, J. O. & Nielsen, L. K. Alleviating monoterpene toxicity using a two-phase extractive fermentation for the bioproduction of jet fuel mixtures in Saccharomyces cerevisiae. *Biotechnol. Bioeng.* **109,** 2513–2522 (2012).](http://paperpile.com/b/3CUptU/JWZXd)

24. [Brennan, T. C. R., Krömer, J. O. & Nielsen, L. K. Physiological and Transcriptional Responses of Saccharomyces cerevisiae to d-Limonene Show Changes to the Cell Wall but Not to the Plasma Membrane. *Appl. Environ. Microbiol.* **79,** 3590–3600 (2013).](http://paperpile.com/b/3CUptU/MUFm2)

25. [Martin, V. J. J., Pitera, D. J., Withers, S. T., Newman, J. D. & Keasling, J. D. Engineering a mevalonate pathway in Escherichia coli for production of terpenoids. *Nat. Biotechnol.* **21,** 796–802 (2003).](http://paperpile.com/b/3CUptU/8zLkL)

26. [Lv, X. *et al.* Dual regulation of cytoplasmic and mitochondrial acetyl-CoA utilization for improved isoprene production in Saccharomyces cerevisiae. *Nat. Commun.* **7,** 12851 (2016).](http://paperpile.com/b/3CUptU/SvdVO)
