# Supplementary material for: Industrial brewing yeast engineered for the production of primary flavor determinants in hopped beer
Source: Nat Commun. 2018 Mar 20;9:965. doi: 10.1038/s41467-018-03293-x (PMC5861129; doi:10.1038/s41467-018-03293-x)
Supplement: Supplementary file 3 — Description of Additional Supplementary Files [file 41467_2018_3293_MOESM3_ESM.pdf]

Description of Additional Supplementary Files:

File Name: Supplementary Dataset 1
Description: Python notebook containing code used to implement mathematical modeling of monoterpene biosynthesis as a function of gene expression.

File Name: Supplementary Dataset 2
Description: Average relative abundance of heterologous proteins in first iteration strains (mean of three biological replicates).

File Name: Supplementary Dataset 3
Description: Data reflection fermentation characteristics of first iteration strains (mean of three biological replicates).
